# Signal Recognition Particle Suppressor Screening Reveals the Regulation of Membrane Protein Targeting by the Translation Rate

**DOI:** 10.1128/mBio.02373-20

**Published:** 2021-01-12

**Authors:** Liuqun Zhao, Yanyan Cui, Gang Fu, Zixiang Xu, Xiaoping Liao, Dawei Zhang

**Affiliations:** a Tianjin Institute of Industrial Biotechnology, Chinese Academy of Sciences, Tianjin, China; b University of Chinese Academy of Sciences, Beijing, China; c Key Laboratory of Systems Microbial Biotechnology, Chinese Academy of Sciences, Tianjin, China; d National Engineering Laboratory for Industrial Enzymes, Chinese Academy of Sciences, Tianjin, China; University of Rochester

**Keywords:** signal recognition particle, suppressor screening, inner membrane protein targeting, translation rate

## Abstract

The signal recognition particle (SRP) is conserved in all living organisms, and it cotranslationally delivers proteins to the inner membrane or endoplasmic reticulum. Recently, SRP loss was found not to be lethal in either the eukaryote Saccharomyces cerevisiae or the prokaryote Streptococcus mutans. In Escherichia coli, the role of SRP in mediating inner membrane protein (IMP) targeting has long been studied. However, the essentiality of SRP remains a controversial topic, partly hindered by the lack of strains in which SRP is completely absent. Here we show that the SRP was nonessential in E. coli by suppressor screening. We identified two classes of extragenic suppressors—two translation initiation factors and a ribosomal protein—all of which are involved in translation initiation. The translation rate and inner membrane proteomic analyses were combined to define the mechanism that compensates for the lack of SRP. The primary factor that contributes to the efficiency of IMP targeting is the extension of the time window for targeting by pausing the initiation of translation, which further reduces translation initiation and elongation rates. Furthermore, we found that easily predictable features in the nascent chain determine the specificity of protein targeting. Our results show why the loss of the SRP pathway does not lead to lethality. We report a new paradigm in which the time delay in translation initiation is beneficial during protein targeting in the absence of SRP.

## Abstract (precis)

Inner membrane proteins (IMPs) are cotranslationally inserted into the inner membrane or endoplasmic reticulum by the signal recognition particle (SRP). Generally, the deletion of SRP can result in protein targeting defects in Escherichia coli.

## INTRODUCTION

Targeting proteins to their proper cellular destination is essential for all cells. The signal recognition particle (SRP)-dependent cotranslational targeting is an efficient and universal way to deliver nascent polypeptides to the membrane. It directs the rapid cotranslational translocation of polypeptide chains, thereby preventing the folding of polypeptides in the cytoplasm (1). Therefore, most inner membrane proteins (IMPs) are targeted to the inner membrane (IM) of prokaryotes and the endoplasmic reticulum of eukaryotes via the SRP pathway (2, 3). SRP is essential for the efficiency and specificity of IMP targeting (4). The efficiency of membrane protein targeting depends mainly on the hydrophobicity of the N-terminal transmembrane domain (TMD). N-terminal TMDs with low hydrophobicity may fail to be recognized by SRP or they may be skipped, and internal hydrophobicity TMDs are selectively recognized by SRP (5, 6). During SRP-dependent targeting, multiple checkpoints allow SRP to discriminate against incorrect substrates. The differences in binding affinity between SRP and cargo proteins is not sufficient to reject incorrect substrates. In the subsequent step, the assembly of the SRP and SRP receptor (SR) closed complex of incorrect substrates is considerably slower than that of correct substrates, which rejects incorrect substrates. Further, the kinetic competition between GTP hydrolysis and cargo uploading increases the fidelity of protein targeting (7). Another important factor that contributes to the specificity in the targeting process is the kinetic competition between the elongation of the nascent polypeptide and the cotranslational targeting to the membrane (8, 9). SRP must bind to its substrate within a limited time window in the crowded cytosol, and this prevents the nascent chains from exceeding a critical length of 140 amino acids (aa) (10, 11). Several studies indicate that the translation elongation rate can modulate protein folding and targeting, and a reduction in translation elongation creates a longer time window for SRP recognition (8, 12, 13). Compared with mammalian SRP, bacterial SRP is devoid of the Alu domain, which arrests translation elongation to prevent protein synthesis before targeting is completed (13). Furthermore, the translation elongation rate of bacteria is higher than that of eukaryotes (14). Thus, translation elongation may play a more crucial role in bacteria than in eukaryotes in the cotranslational targeting process.

SRP is known as a conserved and essential component across all domains. However, two known organisms, the yeast Saccharomyces cerevisiae (15) and the bacterium Streptococcus mutans (16), can survive when the SRP mechanism is disrupted. In S. cerevisiae, the deletion of SRP leads to a dramatic growth defect and accumulation of a substantial fraction of mislocalized SRP-dependent proteins. The reduction in the protein synthesis rate and the induction of heat shock proteins can partly bypass the SRP requirement (17). In S. mutans, the interruption of the *ffh* gene results in acid sensitivity but not the loss of cell viability (16). As in S. cerevisiae, certain physiological responses, including downregulation of protein synthesis and upregulation of chaperones and proteases, protect cells from misfolded or aggregated proteins (18). In addition, the YidC homologue YidC2 in S. mutans may serve as a mechanism to compensate for the lack of SRP (19). Unlike SRP in S. cerevisiae and S. mutans, Escherichia coli SRP is essential for cell viability (20–23). Extensive research has shown that the depletion of SRP in E. coli causes the inefficient targeting of some IMPs, reduction in protein synthesis, and induction of heat shock response. However, these physiological responses cannot explain the essentiality of SRP (22–24). Moreover, a moderate reduction in the concentration of SRP has little effect on cell growth (24). There is no convincing experimental evidence to explain the underlying mechanism of the essentiality of E. coli SRP to date. This is encouraging, as it suggests that the mystery of the essentiality of SRP has not been revealed, and there is a possibility that some IMPs may be relatively independent of SRP. These observations raised the possibility that SRP in E. coli is not essential for cell viability.

Here, we developed an efficient suppressor screening method that enables us to identify SRP suppressors. The suppressor cells could survive when the essential gene *ffh* was deleted (i.e., SRP was nonessential in E. coli). Although the suppressors could bypass the essentiality of SRP in E. coli, the deletion of the SRP mechanism caused severe growth defects and reduced translation efficiency. Furthermore, we found that suppressors can cause translation arrest, which is tuned at the level of translation initiation rather than elongation. We provide evidence that translation initiation pausing can mediate IMP targeting to suppress the loss of SRP.

## RESULTS

### Isolation and characterization of SRP suppressors.

**FIG 1:**
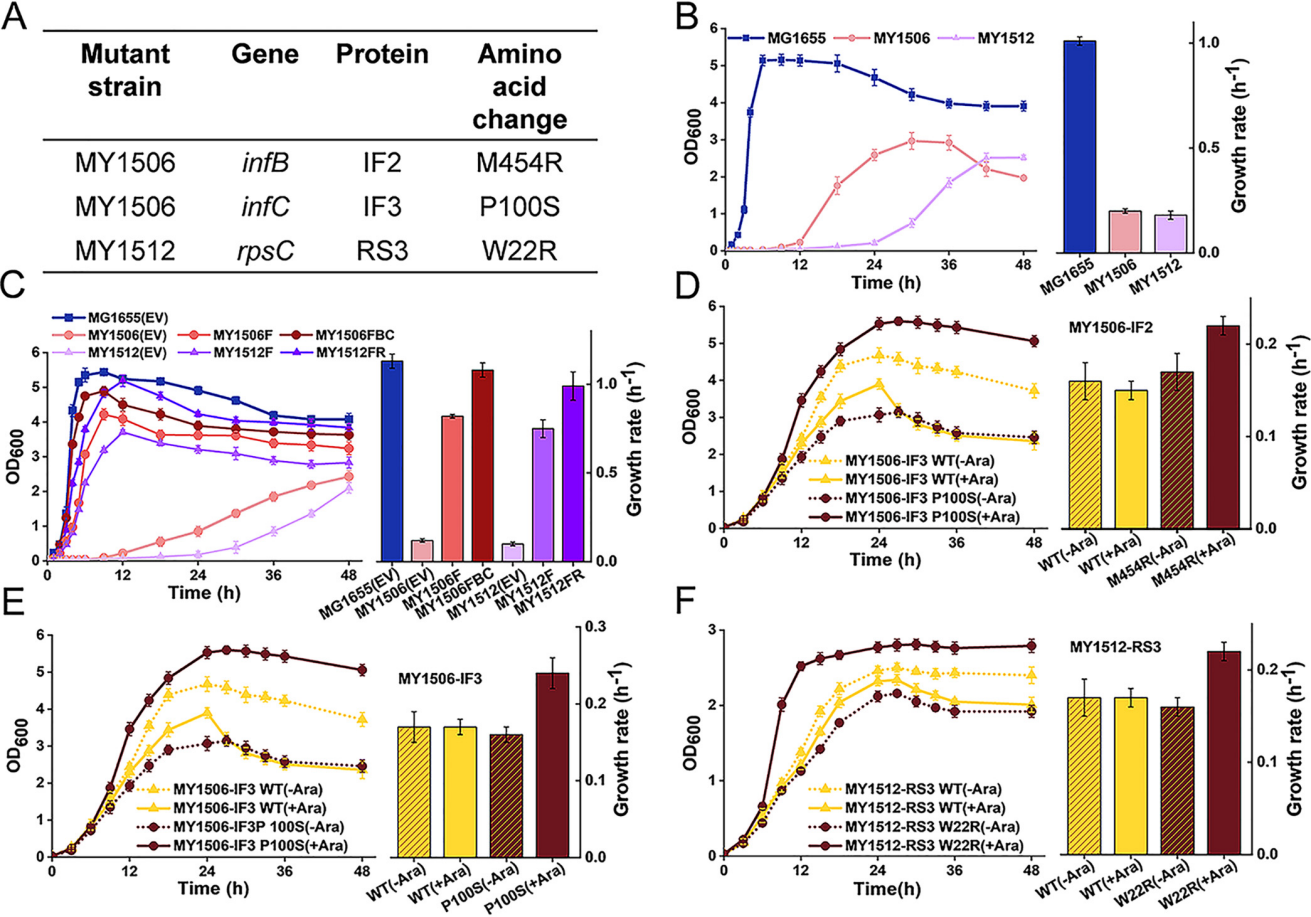
Identified suppressor strains and their growth phenotypes. (A) Suppressors were identified in the genes *infB* and *infC* from the MY1506 strain and in the gene *rpsC* from the MY1512 strain. (B) Growth curves and growth rates of the wild-type strain MG1655 and suppressor strains MY1506 and MY1512. (C) Growth curves and growth rates of revertant cells. MG1655, MY1506, and MY1512 strains containing empty vector (EV) pTrc99K were used as a control group. F, induced expression of Ffh. FBC, suppressor mutations IF2 (*infB*) 454R and IF3 (*infC*) P100S restored to wild-type IF2 and IF3 under the expression of Ffh in the MY1506 strain. FR, suppressor mutation RS3 (*rpsC*) W22R restored to wild-type RS3 under the expression of Ffh in the MY1512 strain. (D to F) Growth curves and growth rates for wild-type and suppressor strains overexpressing wild-type (WT) and mutant suppressor proteins. Protein expression was induced by the addition of arabinose (+Ara). Noninduced cells were cultured without arabinose (–Ara). Solid or short dotted curves are the mean of three independent samples, and error bars represent the standard error of the mean of the samples. Growth rates were calculated from the exponential growth phase. All growth rates shown represent the mean growth rates from three biological replicates, and error bars represent the standard error of the mean.

As deletion of Ffh causes interruptions in the entire SRP pathway and E. coli is unable to survive without the SRP component Ffh (17, 22), it is challenging to obtain a true null *ffh* mutation. We developed a screening method that could screen suppressors of the essential gene *ffh*. Because Ffh depletion causes severe growth defects (22), we first introduced a rescuing plasmid carrying the *ffh* gene into the MG1655 strain, and then deletion of the chromosomal copy of *ffh* was obtained through its replacement with a chloramphenicol acetyltransferase gene (*cat*), yielding strain MY1410 (see Fig. S1A in the supplemental material). Next, the mutagenesis of MY1410 was carried out using atmospheric and room temperature plasma (ARTP) (25) to gain suppressor mutations by losing the rescuing plasmid. After several ARTP mutation treatments and screenings, we obtained several independent colonies that could survive on the screening plates (Fig. S1B). PCR and genome sequencing methods confirmed that both MY1506 and MY1512 completely lost the rescuing plasmid, and the entire chromosomal copy of the *ffh* gene was completely deleted (Fig. S1C). Furthermore, we sequenced the genomic DNA from the two isolated strains and the wild-type (WT) MG1655 strain, and two classes of suppressors were generated: translation initiation factors IF2 and IF3 identified in MY1506 and the ribosomal protein RS3 identified in MY1512 (Fig. 1A). All suppressors are required for translational initiation (26, 27). Three mutations, IF2 M454R, IF3 P100S, and RS3 W22R, were all located in the critical region of each protein (Fig. S1D). The IF2 M454R mutation is in the Switch 2 domain of IF2 that has contacts with GTP and Mg^2+^ and is involved in conformational changes during GTP hydrolysis (28). The IF3 P100S mutation is located in the C-terminal domain of IF3 that binds near the P-site region and engages in the interaction with the 30S subunit (29). The RS3 W22R mutation is in the N-terminal domain of RS3 that is located around the mRNA binding path (30). These results suggest that these mutations may play a critical role in translation initiation.

10.1128/mBio.02373-20.1 FIG S1 Isolation of SRP suppressors. (A) Construction of the MY1410 strain for screening suppressors. A temperature-sensitive plasmid, pKDFB, containing the *ffh* gene and a counterselection marker gene, *sacB*, was used to rescue Ffh deletion cells. (B) Suppressor screening. The cells without suppressors were killed by any of the following conditions: 37°C, 10% (wt/vol) sucrose, and 0.2% (wt/vol) glucose. (C) Agarose gel electrophoresis of colony PCR. Samples from strain MY1410 were used as controls. (D) Localization of suppressors in protein sequences. (E) Plating assay of wild-type MG1655 and candidate suppressor MY1506 and MY1512 strains. Download FIG S1, PDF file, 0.2 MB. Copyright © 2021 Zhao et al. 2021 Zhao et al. This content is distributed under the terms of the Creative Commons Attribution 4.0 International license.

Two candidate suppressor strains, MY1506 and MY1512, survived when the SRP pathway was blocked but showed much slower growth than the wild-type strain MG1655 (Fig. 1B; Fig. S1E). The growth rates of suppressor cells were reduced by approximately 5 times compared with those of wild-type cells (Fig. 1B). To evaluate whether the growth defect was caused by mutations that deleted Ffh or suppressors, we performed a rescue assay by reconstructing the wild-type strain from the suppressor strain. We first expressed Ffh in the suppressor strains and set up a growth assay in these strains. In the absence of the inducer, leaky basal level expression of Ffh in suppressor cells showed an approximately 7 times increase in the growth rate compared with cells carrying the empty vector (Fig. 1C), suggesting that deletion of chromosomal *ffh* had a very severe negative effect on cell growth and was the main reason for the growth defect. Furthermore, with the expression of Ffh, the suppressor mutations in the above-described suppressor strains were changed into the wild-type, which restored the growth rate to nearly the wild-type level (Fig. 1C). Thus, in the presence of SRP, suppressors can exhibit a negative effect on cell growth. To further evaluate the role of suppressors in bypassing the requirement of the SRP pathway, we overexpressed suppressors in the wild-type and suppressor strains. Compared with noninduced cells, the overexpressed cells showed an approximately 1.3-fold increase in the growth rate and a marked increase in the optical density at 600 nm (OD_600_) during the stationary phase in the suppressor strains MY1506 and MY1512 (Fig. 1D to F). In contrast, in the wild-type strain MG1655, multicopy suppressors did not influence the growth rate but significantly decreased the OD_600_ at the beginning of the stationary phase (Fig. S2). To eliminate the possibility that the growth defect could be suppressed by the overexpression of a variety of proteins, we overproduced wild-type IF2, IF3, and RS3 in the MY1506 and MY1512 strains. As expected, the overexpression of these wild-type proteins failed to increase the growth rate or biomass of all test strains (Fig. 1D to F; Fig. S2), which may be due to the increased translation fidelity. Thus, not all multicopy plasmids can bypass the essentiality of SRP, and the suppressors are truly involved in suppressing growth defects when SRP is deleted. Taken together, these results suggest that suppressors are indeed responsible for the complementation of the loss of SRP and that “true” suppressors are obtained.

10.1128/mBio.02373-20.2 FIG S2 Growth assays of MG1655 strain expression of wild-type (WT) and suppressor proteins. Protein expression was induced by the addition of arabinose (+Ara). Noninduced cells were cultured without arabinose (−Ara). (A) Growth curves and growth rates of the MG1655 strain overproducing IF2 WT and suppressor IF2 M454R. (B) Growth curves and growth rates of the MG1655 strain overproducing IF3 WT and suppressor IF3 P100S. (C) Growth curves and growth rates of the MG1655 strain overproducing RS3 WT and suppressor RS3 W22R. The strains had approximately the same growth rate of 1.0 h^−1^, but the OD_600_ of the induced cells decreased during entry into the stationary phase compared with that of the noninduced cells. Solid or short dotted curves represent the mean of three independent samples, and error bars represent the standard errors of the mean of the samples. Growth rates were calculated from the exponential growth phase. All growth rates shown represent the mean growth rates from three biological replicates, and the error bars represent the standard errors of the mean. Download FIG S2, PDF file, 0.2 MB. Copyright © 2021 Zhao et al. 2021 Zhao et al. This content is distributed under the terms of the Creative Commons Attribution 4.0 International license.

### Effects of suppressors on translation efficiency.

**FIG 2:**
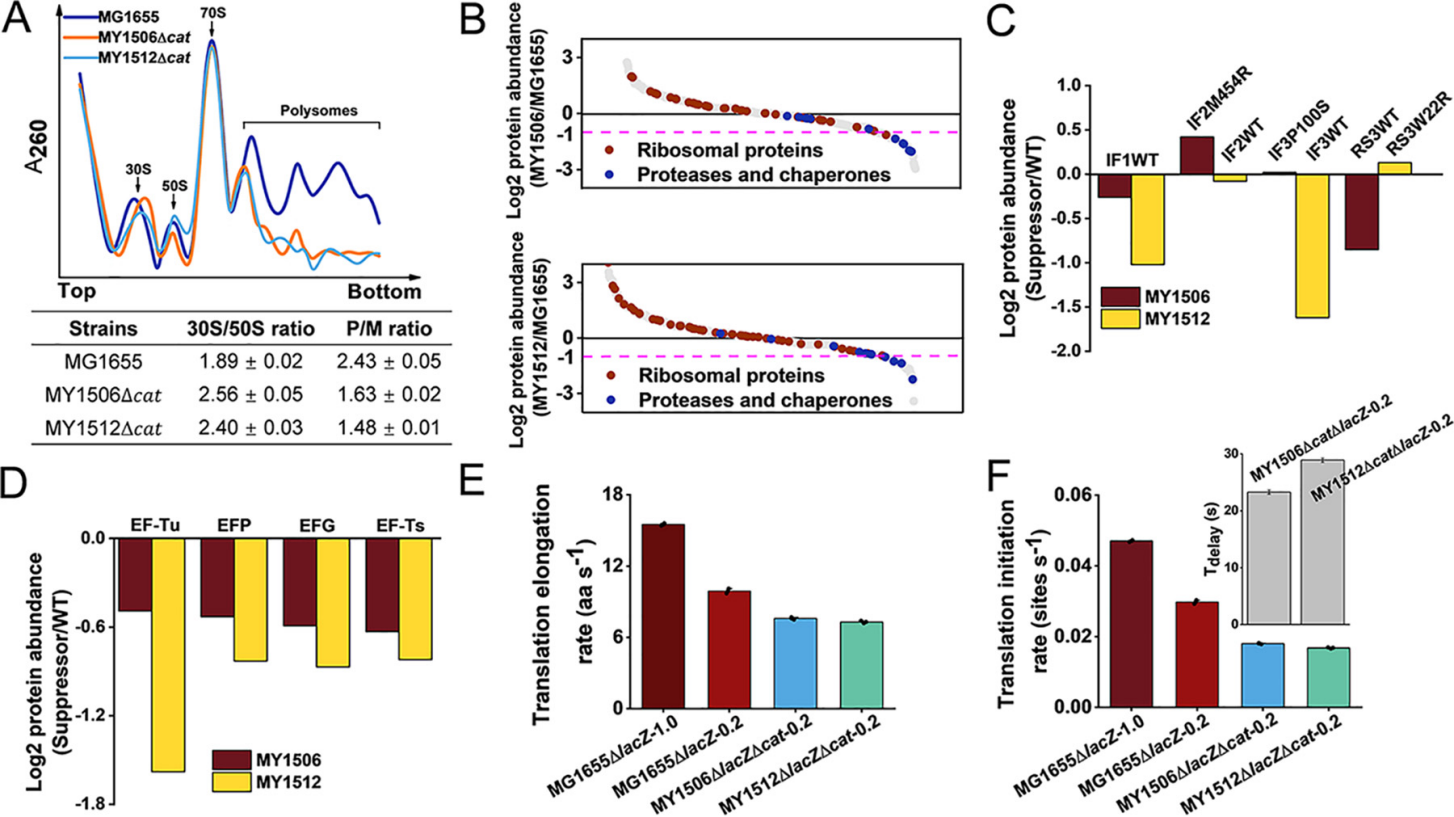
Effects of suppressors on translation efficiency. (A) Polysome profiles of wild-type MG1655 and suppressor MY1506Δ*cat* and MY1512Δ*cat* strains. Data represent the mean ± standard error of the mean of three independent experiments. (B) Quantification of all proteins identified in wild-type MG1655 and suppressor MY1506 and MY1512 strains. The protein abundance of suppressor cells is relative to that of wild-type cells. Ribosomal proteins and proteases and chaperones are indicated. The magenta dashed line indicates half the level of wild-type cells. (C) Log_2_ fold change in the expression of translation initiation factors and ribosomal protein RS3 in suppressor cells relative to those in wild-type cells. (D) Log_2_ fold change in the expression of translation elongation factors in suppressor cells relative to those in wild-type cells. (E) Measurement of translation elongation rates under different growth rates (MG1655Δ*lacZ*, 1.0 h^−1^ and 0.2 h^−1^; MY1506Δ*lacZ*Δ*cat*, 0.2 h^−1^; MY1512Δ*lacZ*Δ*cat*, 0.2 h^−1^). Data are obtained from the induction kinetics assay of the LacZα-fused protein MsbA-LacZα under different MOPS media. Bar heights represent mean values, with error bars indicating standard errors of the mean from three biological replicates. (F) Translation initiation rates of different cells were estimated based on their corresponding translation elongation rates. Inset, time delay (*T*_delay_) in initiation of suppressor cells. Bar heights represent mean values, with error bars indicating standard errors of the mean from three biological replicates.

We next sought to identify the molecular mechanism of cell viability without SRP. Protein translation can modulate membrane protein targeting in the cotranslational translocation pathway (8, 17). To determine whether the translation machinery is impaired to suppress the loss of SRP, we performed a polysome profiling analysis to detect defects of translation. Two suppressor strains showed similar changes in the polysome profiles relative to those of the wild-type strain (Fig. 2A), suggesting that these two classes of suppressors had a similar effect on protein translation. The 30S-to-50S (30S/50S) ratios of suppressor cells were increased by approximately 30% compared with those of the wild-type cells, indicating a deficiency in the assembly of the 50S ribosomal subunit. The polysome-to-70S monosome (P/M) ratios of suppressor cells were decreased to approximately 60% compared with those of wild-type cells, suggesting a reduced protein translation initiation rate, similar to the case of inhibition of translation initiation (31). Surprisingly, the peaks of the 70S ribosome were not altered in the wild-type and suppressor cells, although the assembly of the ribosome was affected. We speculated that some 70S ribosomes may be stalled at the initiation site; thus, the translation initiation rate may be decreased.

To further examine whether suppressors affect the components of the translation machinery, we analyzed the whole-cell proteomes of the wild-type and suppressor strains (Data Set S2A to C). Previous studies have shown that the heat shock response plays a vital role in maintaining cell viability when SRP is inefficient (21, 22, 24). Unexpectedly, heat shock response-related proteases and chaperones were not induced in the suppressor cells. The levels of the representative chaperones DnaK and GroEL dropped by at least 50%. The levels of several vital proteases (ClpB, Lon, and FtsH) were decreased by at least 30% (Fig. 2B). These results suggested that heat shock responses may not be necessary to rescue SRP deletion-induced cell death. Suppressor cells were adapted to the loss of SRP. It is unlikely that the heat shock response plays an important role in protein targeting. Although the repression of ribosomal proteins serves to enhance the efficiency of protein targeting to offset the deficiency of SRP (17, 23), the levels of only a few ribosomal proteins were reduced by 2 times in suppressor cells, and suppressor cells showed increased levels of most of the ribosomal proteins (Fig. 2B). Given this result, we considered that the increased pool of ribosomes may be due to translation inhibition. As predicted, the protein abundances of translation initiation factors in the wild-type (WT) were all decreased. The levels of IF1 WT in strain MY1506 and IF2 WT in strain MY1512 were slightly reduced, and the levels of IF1 WT and IF3 WT in strain MY1512 were decreased by at least 50% (Fig. 2C). In contrast, the protein abundances of translation initiation factors in the suppressor mutation type (IF2 M454R and IF3 P100S) were both modestly increased in the suppressor strains (Fig. 2C). Additionally, the RS3 W22R suppressor in MY1512 was slightly increased, but the protein abundance of wild-type RS3 (RS3 WT) was decreased by approximately 2 times in MY1506 (Fig. 2C). It seems that the upregulation of these suppressors and downregulation of their corresponding wild-type alleles were helpful to suppress the deletion of SRP. Additionally, the expression of elongation factors was reduced by at least 30% in suppressor cells (Fig. 2D), suggesting that the translation elongation rate may also decrease. Thus, based on the proteomic data, the translation initiation and elongation rates were all reduced in the suppressor cells.

However, these results are not sufficient to conclude that the suppressors bypass the requirement of SRP by decreasing the translation rate. Because the elongation rate depends closely on the growth rate (32, 33), we must consider the growth defects of suppressor cells when determining the effects of these suppressors on translation. Therefore, we compared the elongation rates of the wild-type and suppressor cells under the same growth rate (0.2 h^−1^), which was modulated by nutrient limitation (Table S1A). We used a LacZα induction assay to measure the translation elongation rate of cells (33) (Fig. S3B to D). We used the IMP MsbA fused to LacZα to examine the elongation rate. When all cells were grown in the same potassium morpholinopropane sulfonate (MOPS)-rich medium, the growth rate of wild-type cells (1.0 h^−1^) was approximately 5 times higher than that of suppressor cells (0.2 h^−1^) (Table S1A; Fig. S3A), and the growth rates of cells grown in MOPS-rich medium and LB medium were approximately similar (Fig. 1B). The translation elongation rate in wild-type cells (15 aa s^−1^) was approximately 2 times higher than that in suppressor cells (7 aa s^−1^) (Fig. 2E; Fig. S3D). Next, we compared the elongation rates of wild-type and suppressor cells under the same growth rate of 0.2 h^−1^. The elongation rate of wild-type cells (10 aa s^−1^) was still higher than that of suppressor cells (7 aa s^−1^) (Fig. 2E; Fig. S3D). Thus, suppressors could decrease the elongation rate. We also estimated the translation initiation rate by using the homogeneous ribosome flow model (HRFM) (34). The initiation rate of suppressor cells (0.02 sites s^−1^) decreased by 2.5 times compared with that of wild-type cells (0.05 sites s^−1^) at a growth rate of 1.0 h^−1^ and decreased by 1.5 times compared with that of wild-type cells (0.03 sites s^−1^) at 0.2 h^−1^ (Fig. 2F). We also found that the initiation of translation of suppressor cells was delayed for a long time, approximately 26 s, relative to wild-type cells at the growth rate of 0.2 h^−1^ (Fig. 2F; Table S1A). Therefore, suppressors may prevent translation from starting, and this pausing may be the main reason for reduced translation initiation and elongation rates.

10.1128/mBio.02373-20.3 FIG S3 Measurement of the translation elongation rates of wild-type and suppressor cells. (A) Growth curves of strains in different MOPS media. The growth rates of MG1655Δ*lacZ*, MY1506Δ*lacZ*Δ*cat*, and MY1512Δ*lacZ*Δ*cat* grown in glucose plus cAA medium were approximately 1.0 h^−1^, 0.2 h^−1^, and 0.2 h^−1^, respectively. The growth rate of MG1655Δ*lacZ* grown in sorbitol plus NH_4_Cl medium was approximately 0.2 h^−1^ (Table S1A). Solid curves are the mean of three independent biological replicates, and the error bars represent the standard errors of the mean. (B) Calibration of the time cost of initiation steps (*T*_init_) by measuring the induction kinetics of the empty LacZα fragment. (C) Induction curves of the LacZα-fused protein MsbA-LacZα. (D) Schleif plots of the MsbA-LacZα protein plotted against the induction time. Schleif plots were repeated three times, and one typical result shown here. Download FIG S3, PDF file, 0.3 MB. Copyright © 2021 Zhao et al. 2021 Zhao et al. This content is distributed under the terms of the Creative Commons Attribution 4.0 International license.

10.1128/mBio.02373-20.8 TABLE S1 Cell growth in MOPS media and properties of first transmembrane domains. (A) Growth rates of cells under different nutrient conditions. Growth rates were calculated from the corresponding growth curves shown in Fig. S3 and Fig. S4. All values are expressed as the mean ± standard error of the mean. Data shown are related to Fig. 2E and F and Fig. 4A to D. (B) Analysis of first transmembrane domain (TMD1) properties. Data shown are related to Fig. 5E. Download Table S1, PDF file, 0.2 MB. Copyright © 2021 Zhao et al. 2021 Zhao et al. This content is distributed under the terms of the Creative Commons Attribution 4.0 International license.

10.1128/mBio.02373-20.4 FIG S4 Growth curves of cells for protein targeting and translation elongation rate assays. (A to E) Growth curves for the expression of GFP-His_8_-fused proteins in MG1655Δ*lacZ* (A), SRP^+^ strain (B), SRP^−^ strain (C), MY1506Δ*lacZ*Δ*cat* (D), and MY1512Δ*lacZ*Δ*cat* (E). SRP^+^ strain, Ffh expression in the HDB51 strain grown with the addition of arabinose; SRP^−^ strain, Ffh depletion in the HDB51 strain grown with addition of glucose. (F) Growth curves of HDB51 cells in different MOPS media. The growth rate of SRP^+^ cells grown in arabinose plus fructose plus cAA was approximately 1.0 h^−1^. The growth rates of SRP^−^ cells grown in fructose plus cAA medium was approximately 0.2 h^−1^ (Table S1A). (G) Growth curves for expression of GFP-His_8_-fused proteins in SRP^−^ cells at 25°C. (H) Growth curves for the expression of DcuS-GFP-His_8_ in SRP^−^ cells that were grown in LB medium supplemented with 25, 50, and 75 μg ml^−1^ kasugamycin (Ksg). A high concentration of Ksg (75 μg ml^−1^) caused cell death. (I) Growth curves for the expression of GFP-His_8_-fused proteins in SRP^−^ cells grown in LB medium supplemented with 50 μg ml^−1^ Ksg. Solid curves represent the mean of three independent biological replicates, and the error bars represent the standard errors of the mean. Growth rates were calculated from the exponential growth phase. All values are expressed as the mean ± standard error of the mean. Download FIG S4, PDF file, 0.3 MB. Copyright © 2021 Zhao et al. 2021 Zhao et al. This content is distributed under the terms of the Creative Commons Attribution 4.0 International license.

### Suppressors reduce targeting defects of inner membrane proteins.

**FIG 3:**
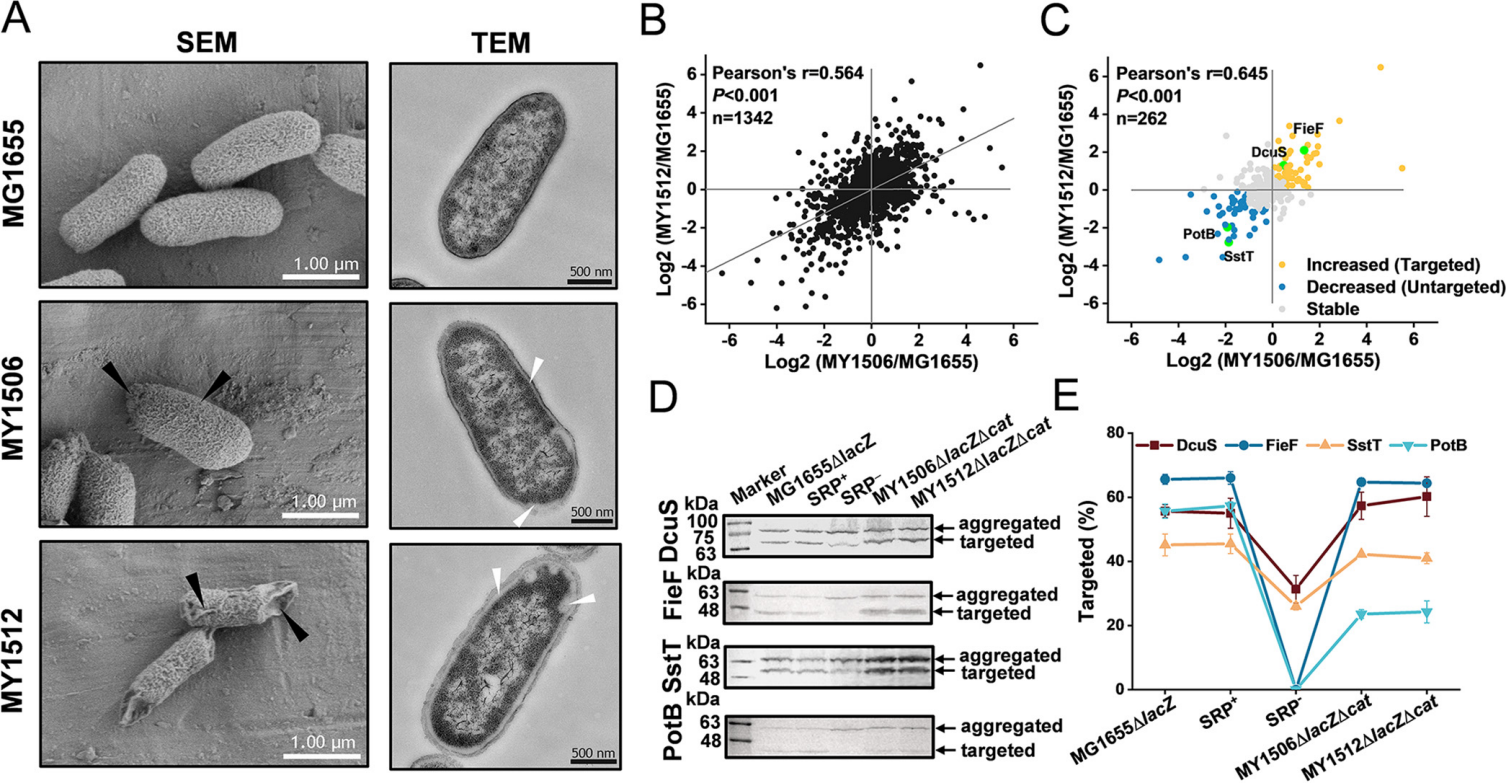
Effects of suppressors on the targeting of inner membrane proteins. (A) Scanning electron microscopy (SEM) and transmission electron microscopy (TEM) analysis of wild-type and suppressor cells. For SEM, the rough and concave surface is indicated by black arrowheads. Scale bar, 1.00 μm. For TEM, the injured inner membrane is indicated by white arrowheads. Scale bar, 500 nM. (B) Full proteome comparison scatterplot of protein abundance changes in MY1506 cells against protein abundance changes in MY1512. Pearson’s *r* and *P* values are indicated. *n* = 1,342 (Data Set S2D). (C) Comparison of targeting changes in SRP-dependent inner membrane proteins based on their protein abundance changes. The inner membrane proteins in suppressor cells are sorted into three classes: “increased,” “decreased,” and “stable.” The increased and decreased classes of proteins were assumed to be targeted and untargeted proteins, respectively. The presumably targeted proteins Dcus and FieF and untargeted proteins SstT and PotB are represented by green dots. Pearson’s *r* and *P* values are indicated. *n* = 262 (Data Set S2E). (D) Detection of targeted and aggregated GFP-fused inner membrane proteins by immunoblotting. (E) Quantification of the percentage of proteins in the targeted state of the total protein (targeted plus aggregated). Data are calculated based on results shown in panel D. Data represent the mean ± standard error of the mean of three independent experiments. SRP^+^, Ffh expression in the HDB51 strain grown with addition of arabinose; SRP^−^, Ffh depletion in the HDB51 strain grown with addition of glucose.

To test whether these suppressors could suppress targeting defects of IMPs, we first examined the integrity of the IM by scanning electron microscopy (SEM) and transmission electron microscopy (TEM). Suppressor strains had the typical rod morphology found in the wild-type strain MG1655 but had moderately damaged membrane structures (Fig. 3A), suggesting that suppressors partially offset the adverse effects of SRP loss on IMP targeting. To further understand the targeting defects of IMPs in suppressor cells, we next investigated the localization of SRP-dependent proteins by the IM proteome. Proteomic changes in the IM of both suppressor cells were measured against those in the wild-type cell. The changes were significantly correlated between strains MY1506 and MY1512 (Fig. 3B). This confirmed that the two suppressor cells had a similar suppression mechanism. The experimental determination of IMPs through proteomic approaches remains challenging because not all IMPs are expressed at levels that are detectable using mass spectrometry (MS) and the extraction of hydrophobic IMPs requires multiple experimental processes that might increase sample loss (35, 36). According to the localization annotations from the STEPdb 2.0 program (37), among the 1,342 identified E. coli proteins, only ∼30% were IMPs (Data Set S2D). We detected 262 IMPs that belong to SRP substrates, according to the cellular substrate pool of the SRP characterized by Schibich et al. (6) (Data Set S2E). We sorted the 262 IMPs into three different classes, namely, “increased,” “decreased,” and “stable” targeting levels that are relative to those in wild-type cells (Fig. 3C; Data Set S2F and G). To better understand the IMP targeting profile, we studied the profoundly different targeting levels of the increased and decreased classes, which were assumed to be “targeted” and “untargeted” protein classes in suppressor cells, respectively. The targeting levels of four IMPs, C_4_-dicarboxylate sensor kinase DcuS (38), zinc transporter FieF (39), serine transporter SstT (40), and spermidine transporter PotB (41), were analyzed by green fluorescent protein (GFP) fusion. IMPs fused with the GFP-His_8_ tag were expressed in cells, allowing the detection of both aggregated and targeted proteins (42). To avoid reaching the level of translocation saturation, the IMP-GFP fusion proteins were expressed with a low concentration of isopropyl β-d-1-thiogalactopyranoside (IPTG; 0.02 mM) (Fig. S4A to E). The wild-type MG1655 and SRP-positive (SRP^+^) strains exhibited similar targeting levels of each analyzed IMP (Fig. 3D and E), which eliminated the possibility that MG1655 and HDB51 had different backgrounds of protein targeting. The targeted levels of all four IMPs (Dcus, FieF, SstT, and PotB) in SRP-negative (SRP^−^) cells were severely negatively affected relative to those in wild-type cells (Fig. 3D and E). For the presumably “targeted” DcuS and FieF proteins, the targeting levels in suppressor cells were nearly equal to those in wild-type cells (Fig. 3D and E), suggesting that the DcuS and FieF proteins could be targeted to the IM. For the presumably “untargeted” protein SstT, the targeting level in suppressor cells was only slightly decreased relative to that in wild-type cells (Fig. 3D and E), indicating that SstT was also qualified as a targeted protein in suppressor cells. This inconsistency may be due to the low expression level of endogenous SstT that may be not detected by MS in suppressor cells. We observed that the PotB protein showed a significantly lower targeting level in suppressor cells than in wild-type cells (Fig. 3D and E), suggesting that PotB is definitely an untargeted protein in suppressor cells. Together with the observation from the IM proteome data, which showed that the degree of the targeting defects varied for different proteins in suppressor cells, these results suggested that these different protein targeting levels may depend on specific properties of these protein sequences. We also found that the targeting levels of the four IMPs in suppressor cells were all higher than those in SRP^−^ cells (Fig. 3D and E), suggesting that suppressors can alleviate the detrimental effects of the loss of SRP on protein targeting. Taken together, our results suggest that a substantial number of IMPs can efficiently target to the IM without SRP and that suppressors can be of importance for this process.

### Translation initiation defects compensate for the inefficiency of protein targeting.

**FIG 4:**
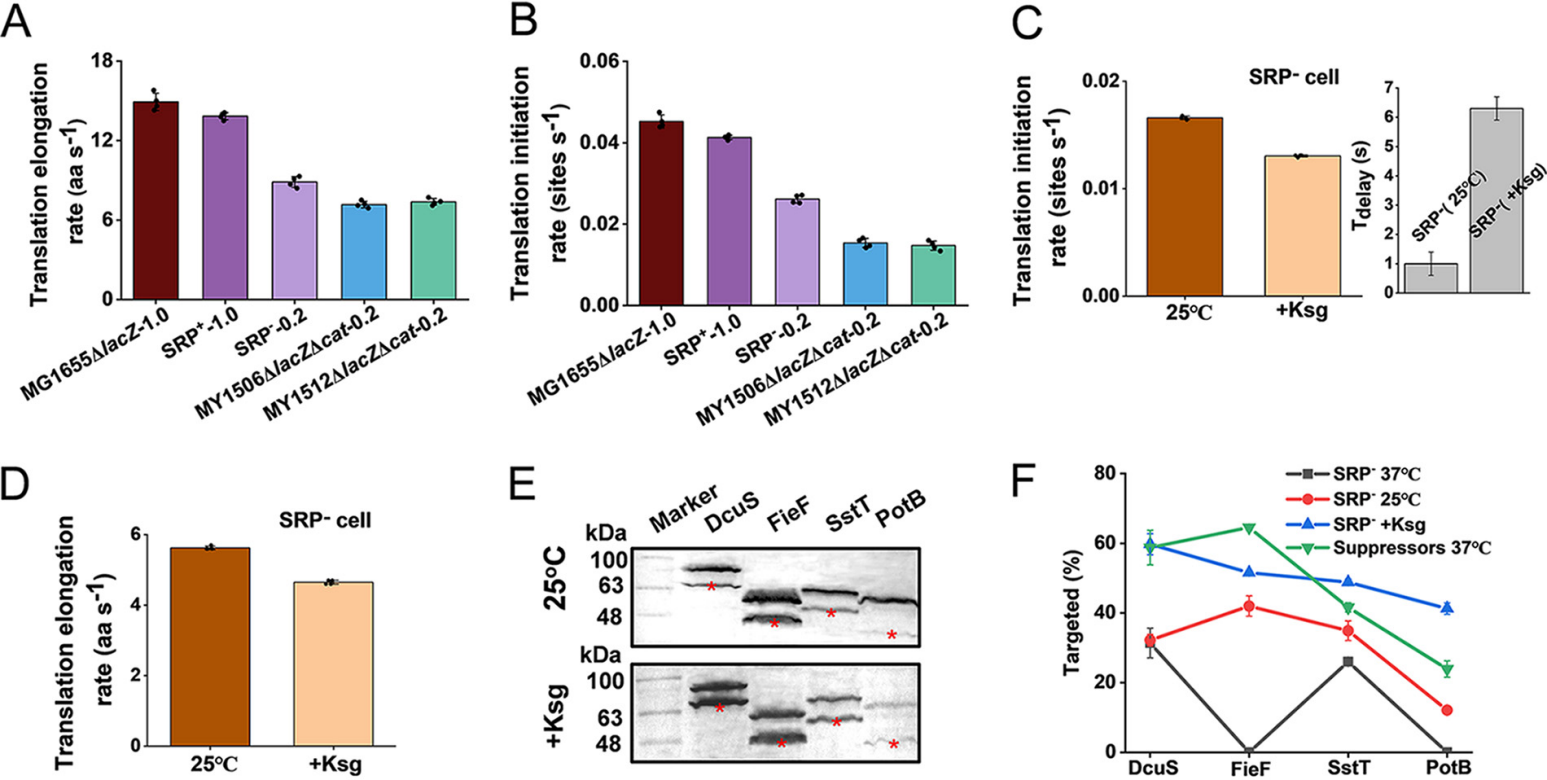
Reduction of translation rates contributes to suppressing targeting defects of inner membrane proteins. (A) Measurement of translation elongation rates under different growth rates (MG1655Δ*lacZ*, 1.0 h^−1^; SRP^+^ strain, 1.0 h^−1^; SRP^−^ strain, 0.2 h^−1^; MY1506Δ*lacZ*Δ*cat*, 0.2 h^−1^; MY1512Δ*lacZ*Δ*cat*, 0.2 h^−1^). SRP^+^ strain, Ffh expression in the HDB51 strain grown with addition of arabinose; SRP^−^ strain, Ffh depletion in the HDB51 strain grown with addition of glucose. Data were obtained from four LacZα-fused proteins, DcuA-LacZα, FieF-LacZα, SstT-LacZα, and PotB-LacZα, under different MOPS media (Table S1A; Fig. S5A to I). Bar heights represent mean values, with error bars indicating standard errors of the mean from the average values of the three biological replicates of these four proteins. (B) Translation initiation rates were estimated based on the translation elongation rates. Bar heights represent mean values, with error bars indicating standard errors of the mean of three biological replicates of each protein. (C) Translation initiation inhibitors, such as growth at 25°C or treatment with kasugamycin (Ksg), decreased initiation. Left, initiation rate of suppressor cells under initiation inhibition; right, relative to growth at 37°C, time delay (*T*_delay_) in initiation of suppressor cells under initiation inhibition. (D) Translation elongation rate of SRP^−^ cells grown at 25°C or treated with Ksg. Bar heights represent mean values, with error bars indicating standard errors of the mean of the three biological replicates of each protein. (E) Immunoblotting analysis of GFP-His_8_-fused proteins in SRP^−^ cells upon inhibition by different translation inhibitors. The targeted fractions are indicated by stars. (F) Quantification of the percentage of proteins in the targeted state of the total protein (targeted plus aggregated). Data are calculated based on the results shown in panel E and Fig. 3D. Data represent the mean ± standard error of the mean of three independent experiments.

To determine whether the decreased translation rate is responsible for IMP targeting in the absence of SRP, we first compared the translation speeds of the four IMPs mentioned above. To eliminate the influence of cell growth on the translation elongation rate, we grew SRP^+^ and SRP^−^ cells in specific MOPS medium to obtain growth rates similar to those of wild-type (1.0 h^−1^) and suppressor (0.2 h^−1^) cells grown in LB medium, respectively (Table S1A; Fig. S4F). In the presence of SRP, the initiation and elongation rates of the SRP^+^ strain were similar to those of MG1655Δ*lacZ* when they grew at the same growth rate (1.0 h^−1^) (Fig. 4A and B; Fig. S4A and B). In the absence of SRP, the elongation rate of the SRP^−^ strain (9 aa s^−1^) was slightly higher than that of suppressor cells (7 aa s^−1^) (Fig. 4A; Fig. S5A to I). The initiation rate of SRP^−^ cells (0.03 sites s^−1^) was approximately 1.5 times higher than that of suppressor cells (0.02 sites s^−1^) (Fig. 4B). These results led to the belief that a reduction in both the initiation and elongation rates contributed to suppressing the loss of SRP. Suppressors are involved in translation initiation, and initiation plays an important role in determining the efficiency of elongation (43, 44). We speculated that initiation is the regulatory step of translation and protein targeting.

10.1128/mBio.02373-20.5 FIG S5 Measurement of translational elongation rates and the correlations between initiation and elongation in different cells. (A) Calibration of the time cost of initiation steps (*T*_init_) of different cells by measuring the induction kinetics of the empty LacZα fragment. (B to E) Induction kinetics of the DcuS-LacZα protein (B), FieF-LacZα protein (C), SstT-LacZα protein (D), and PotB-LacZα protein (E). The MG1655Δ*lacZ*, MY1506Δ*lacZ*Δ*cat*, MY1512Δ*lacZ*Δ*cat*, and SRP^+^ strains were grown at 37°C. The SRP^−^ strain was grown at 37°C or 25°C and treated with kasugamycin (Ksg). (F to I) Schleif plot of the DcuS-LacZα protein (F), FieF-LacZα protein (G), SstT-LacZα protein (H), and PotB-LacZα protein (I). The Schleif plots in panels F to I were repeated three times, and one typical result is shown here. (J) Significant correlation existed between translation initiation and elongation in different cells. After the removal of suppressor cells, this correlation was stronger. Download FIG S5, PDF file, 0.4 MB. Copyright © 2021 Zhao et al. 2021 Zhao et al. This content is distributed under the terms of the Creative Commons Attribution 4.0 International license.

To further understand the targeting process affected by translation in suppressor cells, we tried to mimic the suppressor cells in the SRP^−^ strain by reducing the translation rate. Because suppressors were directly associated with translation initiation, we first grew cells at 25°C (Fig. S4G), which is known to decrease the translation initiation rate (45), and we found that growth at 25°C decreased both translation initiation and elongation rates. The initiation rate (0.02 sites s^−1^) and elongation rate (6 aa s^−1^) of SRP^−^ cells grown at 25°C were reduced by 1.5 times relative to those of cells grown at 37°C (Fig. 4A to D; Fig. S5A to I). We found that the targeting efficiency of the DcuS protein at 25°C was approximately equal to that at 37°C, but the targeting levels of the other three proteins were elevated by at least 34% relative to those at 37°C (Fig. 4E and F). These results implied that growth at 25°C improved membrane protein targeting by decreasing translation initiation and elongation rates. We speculated that further decreases in initiation and elongation rates may result in higher targeting levels of IMPs. We used kasugamycin (Ksg), a well-known translational initiation blocker, to inhibit translation initiation by preventing the association between the 30S and 50S subunits (46), which decreased both translation initiation and elongation rates (Fig. 4C and D). SRP^−^ cells were grown in LB medium with a sublethal dose of Ksg (50 μg ml^−1^) (Fig. S4H and I). Relative to SRP^−^ cells grown without Ksg, the initiation rate (0.01 sites s^−1^) and elongation rate (5 aa s^−1^) were reduced by 3 and 1.8 times, respectively (Fig. 4A to D). Relative to suppressor cells, the initiation and elongation rates were reduced by 2 and 1.4 times, respectively (Fig. 4A to D; Fig. S5A to I). Thus, the initiation and elongation rates of cells treated with Ksg were all further decreased compared to those of cells grown without Ksg at 25°C. We also found that SRP^−^ cells treated with Ksg showed a longer time delay (Fig. 4C; Table S1A), suggesting that the time delay of initiation may assist in protein targeting. As expected, the addition of Ksg to cells profoundly increased the efficiency of IMP targeting compared to the efficiency of cells grown without Ksg at 25°C (Fig. 4E and F). The targeting level of all four proteins grown in SRP^−^ cells with Ksg was elevated at least 1.9 times compared to that in SRP^−^ cells grown without Ksg. The targeting level of the DcuS protein in SRP^−^ cells with Ksg was similar to that in suppressor cells (Fig. 4E and F). Compared with suppressor cells, the targeting levels of the SstT and PotB proteins were increased in SRP^−^ cells grown with Ksg. Although the targeting level of FieF was still low, FieF could target to the IM when SRP^−^ cells were treated with Ksg (Fig. 4E and F). Thus, translation inhibition is an effective strategy for IMP targeting in SRP-blocking cells. These results suggested that increasing the time delays in translation initiation and then decreasing translation initiation and elongation rates can enhance protein targeting. We also found a significant correlation between the translation initiation rate and elongation rate in all cells (Fig. S5J), which is consistent with the belief that the translation initiation rate is significantly correlated with the elongation rate (43). However, this correlation in suppressor cells did not completely match that in other cells (Fig. S5J). This difference may be caused by the very long time delays in initiation (Fig. 2F), which can make the initiation rate decrease and deviate from generally applicable trajectories. Taken together, the time delays in translation initiation can decrease the translation rate, which alleviates the defects of IMP targeting.

### Determinants of the protein targeting specificity in nascent chains.

**FIG 5:**
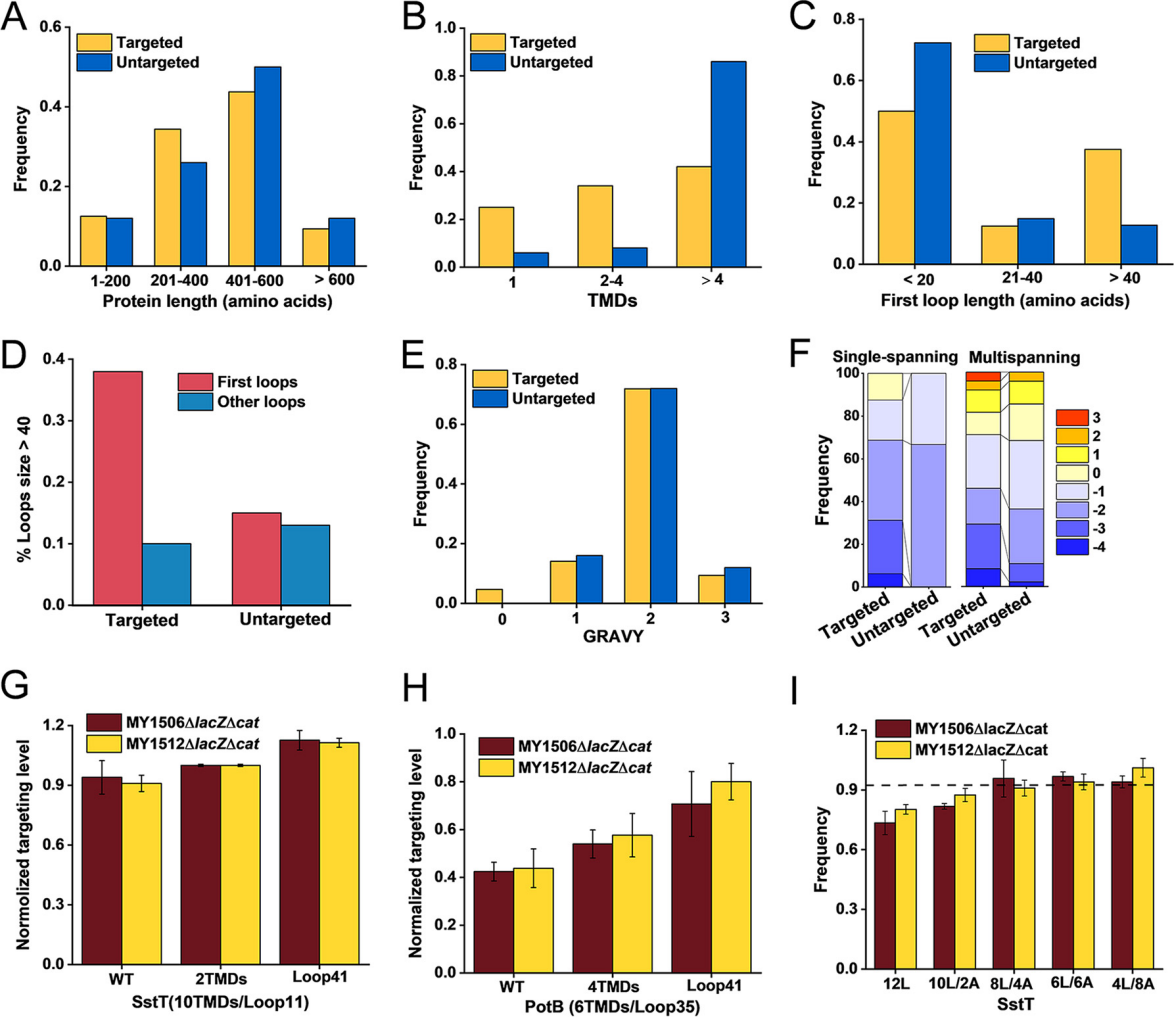
Determinants of the protein targeting specificity in nascent chains. (A to C) Histogram showing the distribution of the protein length (A), the number of TMDs (B), and the length of first loops (C) of the assumed “targeted” and “untargeted” proteins in suppressor cells. (D) Histogram showing the distribution of long loops of >40 aa in first loops or other loops. (E) Histogram showing the distribution of the grand average of hydropathy (GRAVY) value of TMD1 of targeted and untargeted proteins in suppressor cells. (F) Quantification of the computed average Gibbs free energy (Δ*G*_app_) of targeted and untargeted proteins in suppressor cells. (G and H) Quantification of targeting levels of SstT variants (G) and PotB variants (H) in suppressor cells. (I) Effect of hydrophobicity or Δ*G*_app_ value of TMD1 of SstT on protein targeting. A dashed line indicates the average targeting level of wild-type SstT in suppressor cells. The details of variants from panels G to I are shown in Fig. S6 in the supplemental material. The protein targeting levels in suppressor cells are normalized to those in wild-type cells. Error bars represent standard errors of the mean from three independent experiments.

The targeting levels varied among different proteins in suppressor cells, implying that the variable nascent chain sequence may contribute to specific targeting. To understand the determinants that confer specific targeting of SRP-dependent proteins, we compared the properties of the most extreme classes, the “targeted” and “untargeted” protein classes in suppressor cells. The protein lengths of the two classes were similar (Fig. 5A), suggesting that protein length may not be the factor affecting the specificity of targeting. The untargeted class was strongly enriched for multispanning proteins (Fig. 5B) that would be easily misfolded in the cytoplasm when the SRP was lost. However, approximately 40% of the targeted proteins were still successfully targeted to the IM when the number of TMDs surpassed four (Fig. 5B), suggesting that there were other factors related to the specificity of protein targeting. Since multispanning proteins tend to aggregate in the cytoplasm, they have a narrow time window for membrane targeting. If the loops between the TMDs are sufficiently long, the interplay between these hydrophobic TMDs would be attenuated. Our analysis revealed that longer loops were enriched at the first loop between the first TMD (TMD1) and the second TMD (TMD2) of targeted proteins but not at other positions (Fig. 5C and D). Approximately 70% of untargeted proteins in suppressor cells had short first loop lengths of ≤20 residues (Fig. 5C). Thus, a longer first loop would facilitate nascent peptide targeting, which is in agreement with a recent study (47). This result also confirms that the classical model of membrane targeting is mediated mainly by the N-terminal sequence (48). We next analyzed the amino acid sequence of TMD1, which is crucial for IMPs targeted to the IM. The amino acid composition and grand average of hydropathy (GRAVY) value (49) of TMD1s were not significantly different in suppressor cells (Fig. 5E; Table S1B), suggesting that the residues and average hydrophobicity of TMD1s may not contribute to the different levels of protein targeting. We also compared the average Gibbs free energy (Δ*G*_app_) values that are associated with the insertion of TMDs from the translocon to the IM and that varied with the positions, lengths, and amphiphilicities of residues in the TMDs (50). Because the identified single-spanning proteins of the two classes belong mostly to the targeted class and the single-spanning proteins had lower Δ*G*_app_ values than multispanning proteins (51), we further divided each class into single-spanning and multispanning classes to rectify this comparison. The TMD1s of the targeted class proteins had a Δ*G*_app_ value similar to that of the untargeted class proteins (Fig. 5F), suggesting that the Δ*G*_app_ may not be the influencer for protein targeting. We also found that the Δ*G*_app_ value of TMD1s for most proteins was sufficiently low to spontaneously insert into the membrane (Δ*G*_app_ < 0 kcal mol^−1^) (Fig. 5F) (50). Together, these results suggest that the residues within TMD1 may not be involved in the specificity of protein targeting; rather, the number of TMDs and the length of the first loop of IMPs contribute to this specificity.

10.1128/mBio.02373-20.6 FIG S6 Immunoblotting analysis of several membrane protein variants. (A and B) Detection of targeted and aggregated GFP-fused SstT (A) and PotB (B) variants. Left panel, secondary structures of wild-type and mutant proteins; right panel, immunoblotting. (C) Detection of targeted and aggregated GFP-fused SstT variants in which TMD1 had different hydrophobicity or Δ*G*_app_ values. Left panel, secondary structures of wild-type and mutant proteins. The grand averages of hydropathy (GRAVY) and Δ*G*_app_ values are shown. Right panel, immunoblotting. Download FIG S6, PDF file, 0.3 MB. Copyright © 2021 Zhao et al. 2021 Zhao et al. This content is distributed under the terms of the Creative Commons Attribution 4.0 International license.

Next, we constructed a series of membrane protein variants to confirm the above estimation (Fig. S6A and B). Because it is difficult to discriminate whether the mutations directly affect protein targeting or protein structure stability, the targeting level of these mutant proteins in suppressor cells was normalized by the corresponding targeting level in wild-type cells. The truncated protein SstT-2TMDs lacking 8 TMDs at the C terminus of the SstT protein showed a slightly increased targeting level compared with that of the wild-type SstT protein (Fig. 5G; Fig. S6A). The truncated protein PotB-4TMDs lacking 2 TMDs at the C terminus of the PotB protein exhibited an increased targeting level by nearly 10% relative to that of the wild-type PotB protein (Fig. 5H; Fig. S6B). Thus, fewer TMDs are beneficial for protein targeting in SRP-deleted cells with suppressors, because they are less prone to misfolding and aggregation. We also enlarged the first loop length of the SstT and PotB proteins, and the targeting level of the SstT-loop41 and PotB-loop41 variants increased by approximately 20% and 30% relative to the wild-type proteins (SstT-WT and PotB-WT), respectively (Fig. 5G and H; Fig. S6A and B). Thus, a longer first loop was responsible for protein targeting because the interaction of adjacent TMDs would be attenuated to prohibit protein aggregation, and the N-terminal sequence may be allowed to be longer before it loses the capability to target to the IM. We speculated that longer first loops may provide more time for nascent peptides to be targeted to the membrane. We also examined whether the hydrophobicity or Δ*G*_app_ value would affect protein targeting. The hydrophobicity or Δ*G*_app_ value of TMD1 of SstT was changed by replacing certain amino acids with leucine and alanine (Fig. S6C). As expected, none of these varied TMD1 sequences significantly affected the protein targeting level (Fig. 5I). However, the extremely hydrophobic TMD1s, 12L and 10L/2A, showed a slightly decreased targeting efficiency relative to that of other variants (Fig. 5I), suggesting that without SRP, the very strongly hydrophobic TMD1 may have difficulty targeting IMPs to the IM due to its strict dependency on SRP. Taken together, these results indicate that the difference in the TMD1 sequences in targeted and untargeted proteins is minor; however, the number of TMDs and the first loop length in the two classes of proteins were markedly different, which may contribute to the specificity of protein targeting.

## DISCUSSION

The SRP-dependent pathway has been known for nearly 4 decades, but the essentiality of SRP in organisms is still not well understood. Our work revealed that as in S. cerevisiae and S. mutans, SRP is also nonessential in E. coli. Proteomic and experimental analyses suggested that these suppressors of SRP can inhibit translation (Fig. 2C to F; Fig. 4A and B), indicating that they exert their effect on protein synthesis rather than on protein targeting. Compensation for the defects in protein translocation by decreasing protein synthesis caused by physiological responses has been observed previously. In E. coli, suppressor mutations involved in protein synthesis can compensate for the translocation defects of the Sec machinery (52, 53). In S. cerevisiae and S. mutans, physiological responses such as reduced protein synthesis and induction of heat shock proteins contribute to suppressing the loss of SRP (17, 18). In this work, we favored the possibility that suppressors reduced the efficiency of translation due to suppressor mutations. The suppressor strains were in an adapted state without the upregulation of heat shock-regulated proteins (Fig. 2B), and the multicopy suppressors and upregulation of suppressors significantly improved cell growth (Fig. 1D to F; Fig. 2C), confirming that suppressors play a critical role in suppressing the loss of SRP. Hence, we first identified the suppressors of SRP in E. coli, and the essentiality of SRP can be bypassed.

A previous study suggested that reduced cell growth can enhance the fidelity of protein targeting in yeast (17). Although our results showed that SRP inefficiency caused severe growth defects, global growth defects were not sufficient to explain protein targeting without SRP in E. coli. In our work, the growth rates of SRP^−^ cells grown at 37°C and 25°C were similar, but the protein targeting levels were different (Fig. 4F; Fig. S4F and G), suggesting that reducing the growth rate alone could not compensate for the SRP deletion. Thus, the suppression of protein targeting defects in SRP deletion cells was not caused simply by growth defects in E. coli.

**FIG 6:**
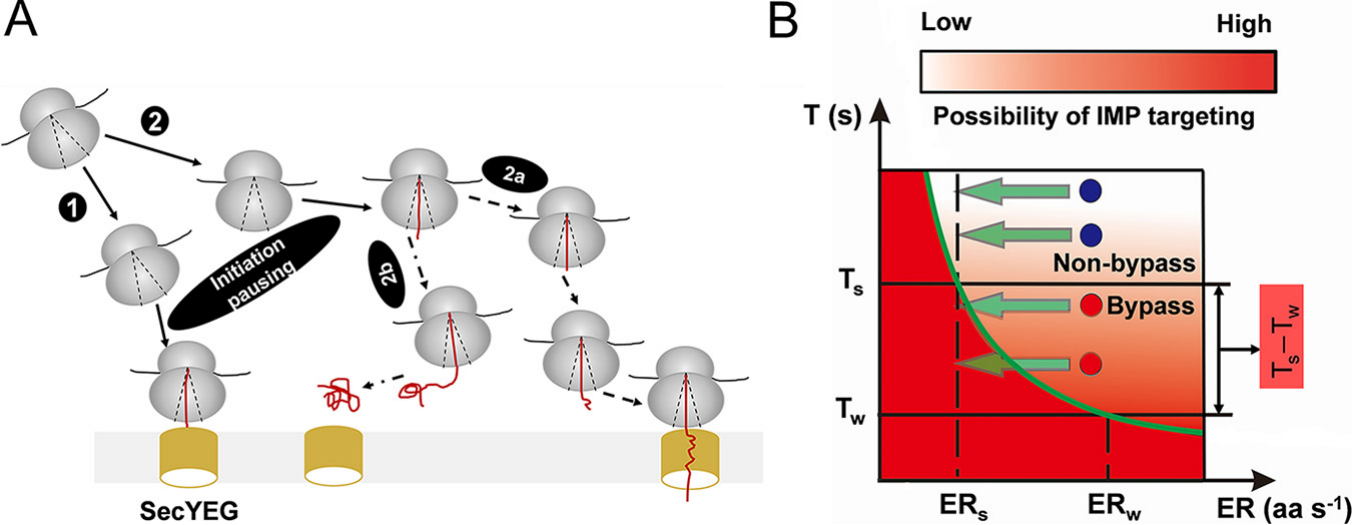
Mechanism of IMP targeting with the aid of SRP suppressors. (A) Schematic depiction of two targeting pathways for delivering IMPs to the IM. The ribosome-bound mRNAs would be targeted to IM either via complete diffusion (route 1) or by combining mRNA diffusion and decreasing the elongation rate of the translating ribosome-mRNA complex (route 2). Suppressors can arrest translation initiation, and this initiation pausing provides a wider time window for enhancing protein targeting. If the mRNA fails to successfully localize to the IM during the delay time, a reduction in the elongation rate that results from the inhibition of initiation would further enlarge the time window for protein targeting (oval labeled 2a). If the compensatory mechanisms cannot deliver IMPs to the translocon SecYEG, the IMPs would misfold and aggregate in the cytoplasm (oval labeled 2b). (B) Relationship between the translation elongation rate (ER) and protein targeting time (*T*). Only when ER times *T* was less than or equal to *L* (critical length) could nascent peptides successfully target to the membrane. In wild-type cells, protein targeting is fast, and the time for targeting (*T_w_*) is short, allowing cells to translate at a high elongation rate (ER*_w_*). Suppressor cells without SRP require a longer time for targeting, and the translation elongation rate of these cells (ER*_s_*) must be reduced. Different proteins require different time windows to bypass SRP. If the time for targeting of some membrane proteins (red circles) is shorter than *T_s_*, these proteins could reach the targeting threshold (green curve). For some membrane proteins (dark blue circles) that have longer targeting times than *T_s_*, a reduction in the elongation rate at ER*_s_* could not target these proteins. Suppressors can extend the upper limit of the critical time window from *T_w_* to *T_s_* and gain extra time (*T_s_* – *T_w_*) to achieve membrane protein targeting. For details and references, see Discussion.

In E. coli, mRNAs encoding IMPs are considered to reach the IM in a translation- and SRP-dependent manner (54, 55) or via a mechanism in which the ribosome-mRNA complex can localize to the IM independently of translation (56) or by combining the two different pathways (57). We found that the SRP suppressors not only decreased the translation rate (Fig. 4A and B) but also delayed the initiation of translation (Fig. 2F), which may be caused by the stalled initiation complex. Theoretically, the time delay might contribute only to translation-independent mRNA reaching the IM. The time delay may be necessary and sufficient for some mRNA targeting to the membrane (Fig. 6A). However, our experiments show that when the dwell time of initiation of translation of SRP^−^ cells is shorter than that of suppressor cells, the translation speed needs to be lower than that of suppressor cells to localize nascent peptide. This implies that the targeting of IMPs may go through two phases: first, the ribosome-mRNAs diffuse close to the IM, and second, the translation initiates, and the lowered translation rate helps localize them (Fig. 6A). The translation initiation pausing can decrease the translation rate. Thus, the time delay in initiation is the primary factor that contributes to the localization of translation-dependent mRNAs by reducing the exposure time of the ribosome-mRNA complex in the cytoplasm. Previously, evidence of translation elongation regulating translating IMP targeting has emerged (8, 9). In our experiment, suppressors directly inhibited translation initiation, which minimized the potential of a ribosome traffic jam, thus minimizing the cost of protein translation (44). Taken together, the SRP suppressors can modulate the targeting of untranslated and translating IMP mRNAs. It is better to set the regulatory point at the translation initiation than at the elongation.

Although suppressors can make a substantial number of IMPs target to the IM, the targeting level of different IMPs was varied in suppressor cells (Fig. 3C). In addition to the diffusion of mRNA before initiation, another influencer is the kinetic competition between elongation and targeting after initiation (8, 9). Without SRP, the targeting rate of IMPs should decrease; thus, if the IMPs need to be targeted to the IM, the translation elongation rate of those proteins must also decrease, which is consistent with the results of lowering the elongation rate in suppressor cells (Fig. 2E). We assumed that the critical length for a specific class of protein is constant, and when at a specific elongation rate, the critical time window for one protein targeting to the IM is constant (Fig. 6B). With SRP in wild-type cells, protein targeting may take less time than the upper limit (*T_w_*) of the critical time window. In suppressor cells, the elongation rate is low, and the upper limit (*T*_i_) of the time window for protein targeting is higher than *T_w_*. Thus, a lowered elongation rate could make some IMPs with a shorter targeting time than *T_s_* efficiently target to the IM and bypass the SRP requirement. If the targeting time of some IMPs is longer than *T_s_*, these proteins would not be localized to the IM and would not bypass SRP essentiality (Fig. 6B). This model suggests that lowering the elongation rate can extend the upper limit of the critical time window from *T_w_* to *T_s_*, and the extra time (*T_s_* – *T_w_*) obtained would increase the possibility of the proper localization of IMPs. Moreover, we also found that proteins with fewer TMDs and longer first loops were more easily targeted (Fig. 5B and C), which may benefit from having longer critical lengths. This suggests that the extended critical length of IMPs can directly enlarge the critical time window of targeting. Because these parameters are easily analyzed from sequences, our results suggest a convenient way to estimate the protein targeting level from only the protein sequence when SRP is inefficient.

SRP suppressors were mapped to chromosomal sites that affect protein translation but not to chromosomal sites involved in protein translocation. This ensures that it is the decreasing translation rate and not various protein translocation chaperones that plays a primary role in membrane protein targeting in the absence of SRP. Previous studies have shown that SecA contributes to the cotranslational translocation of a subset of IMPs in bacteria (58–60). We found that the expression level of SecA was increased by approximately 2-fold in suppressor cells (Fig. S7), suggesting that SecA may cotranslationally target some SRP substrates to the IM when SRP is inefficient. In bacteria, SRP-dependent targeting and SecA-dependent targeting are two separate mechanisms, and their low fidelity in recognizing substrates results in protein mistargeting and aggregation (61, 62). Thus, the elevated SecA expression is not a major factor in allowing cell survival upon SRP deletion. Additionally, the levels of the holo-translocon SecYEG-SecDF-YajC-YidC assisting in membrane protein insertion (63) and the SRP receptor (FtsY) binding to SRP (64) were not markedly changed (Fig. S7; Data Set S2F), suggesting that the components of the transport machinery may play limited but not necessary roles in IMP targeting without SRP. Given that the highly hydrophobic TMD1 is sufficient for spontaneous insertion into the membrane without chaperone assistance (Fig. 5F) and that the more easily aggregated multispanning proteins become more difficult to localize properly (Fig. 5G and H), we speculated that proteins may autonomously target to the membrane within the limited time window. Considering that strongly hydrophobic IMPs are easily aggregated in the cytoplasm (3), it seems unlikely that IMPs localize to the membrane through the posttranslational targeting pathway after completion of the synthesis. We conclude that IMPs can be cotranslationally targeted to the membrane without SRP by decreasing the translation rate to extend the targeting time window.

10.1128/mBio.02373-20.7 FIG S7 Fold change in the expression of transporter genes in suppressor cells relative to those in wild-type cells. A dashed line indicates the expression level of proteins in wild-type cells. Download FIG S7, PDF file, 0.1 MB. Copyright © 2021 Zhao et al. 2021 Zhao et al. This content is distributed under the terms of the Creative Commons Attribution 4.0 International license.

## MATERIALS AND METHODS

### Strains, plasmids, and growth conditions.

Bacterial strains and plasmids used in this study are listed in Data Set S1A in the supplemental material. Primers used for the construction of strains and plasmids are listed in Data Set S1B. E. coli MG1655 was used as the wild-type strain. The Ffh depletion strain HDB51 is a derivative of E. coli WAM113 in which the expression of *ffh* was induced by the *araBAD* promoter (23, 65). All plasmids were constructed either by restriction enzyme digestion and ligation cloning (66) or directly by Gibson assembly (67). Unless otherwise noted, E. coli strains were grown either in Luria broth (LB) medium or on LB agar at 37°C. When necessary, LB was supplemented with 100 μg ml^−1^ ampicillin, 20 μg ml^−1^ chloramphenicol, or 10 μg ml^−1^ gentamicin.

10.1128/mBio.02373-20.9 DATA SET S1 (A) Strains and plasmids used in this study. (B) Primers used in this study. Download Data Set S1, XLSX file, 0.2 MB. Copyright © 2021 Zhao et al. 2021 Zhao et al. This content is distributed under the terms of the Creative Commons Attribution 4.0 International license.

10.1128/mBio.02373-20.10 DATA SET S2 Protein profiles of whole-cell lysates and inner membrane of wild-type and suppressor cells. (A) Proteins identified in the whole-cell lysates of MY1506. (B) Proteins identified in the whole-cell lysates of MY1512. (C) Log2 fold change profile of specific proteins in the whole-cell lysates of suppressor cells versus wild-type cells. (D) Inner membrane proteins quantified by SWATH. (E) Identification of SRP substrates from the inner membrane proteomic analysis. (F) The group of “increased” proteins. (G) The group of “decreased” proteins. (H) Fold change of differential expression of transporter genes in suppressor cells. Download Data Set S2, XLSX file, 0.4 MB. Copyright © 2021 Zhao et al. 2021 Zhao et al. This content is distributed under the terms of the Creative Commons Attribution 4.0 International license.

### Isolation and validation of suppressors.

To generate suppressor mutants, we constructed strain MY1410, which was used as the background strain for screening SRP suppressors. Deletion of the *ffh* gene in E. coli can cause severe growth defects (20). The temperature-sensitive plasmid pKDFB, carrying the negative selection marker *sacB*, which is sensitive to sucrose (68), was used to express *ffh* under the *araBAD* promoter. Then, the plasmid pKDFB was transformed into MG1655. The full *ffh* coding region on the chromosome was then replaced with a chloramphenicol acetyltransferase gene (*cat*) gene. A mutant library was constructed by ARTP mutagenesis, which can cause great DNA damage to individual living cells, the mutation rate of which was also high (25). For mutation, the MY1410 strain was incubated in LB medium supplemented with 0.2% arabinose. When the OD_600_ reached 0.5, the cells were collected and washed with sterilized 0.9% saline. Ten microliters of cell suspension was then dipped onto a sterilized steel plate. The plate was treated for 45 s by the helium ARTP at a gas flow rate of 10.0 standard liters per min (slpm) and a 100 W radiofrequency power input, which caused approximately 90% death. For screening suppressors, the mutated cells were cultivated on solid LB containing 10% sucrose and 0.2% glucose at 37°C for 5 days. Sucrose (10%) was used for *sacB* gene-based counterselection, and glucose (0.2%) was used for repression of the *araBAD* promoter. At 37°C, plasmid pKDFB could be lost in the MY1410 strain. These three screening conditions make the strain under a background of complete deletion of the *ffh* gene. Each surviving colony was picked up and cultivated on LB plates for rescreening. Mutations of the suppressor strains were identified by whole-genome DNA sequencing. To confirm the mutations, PCR amplification of the affected region with locus-specific primers and DNA sequencing were used.

### Growth assays.

Bacterial growth rate was estimated by a growth curve assay. Overnight cultures were diluted in 30 ml fresh LB medium starting from an OD_600_ of ∼0.02 and were grown at 37°C with shaking, at 220 rpm. The OD_600_ was measured at several time points during growth. Growth curves were generated and used to calculate the growth rates. Growth was measured from three biologically independent samples. Error bars represent the standard error of the mean. For the agar plating assay, E. coli strains MG1655, MY1506, and MY1512 were grown into the early exponential phase and diluted to an OD_600_ of 1.0 in LB medium. Ten-fold dilutions of bacterial cultures were spotted onto LB agar plates. The MG1655 strain was incubated for 18 h at 37°C. The MY1506 and MY1512 strains were incubated for 24 h at 37°C.

### Cell morphology and ultrastructure.

The scanning electron microscopy (SEM) and transmission electron microscopy (TEM) assays were used to observe the morphology and integrity of the inner membrane in E. coli MG1655, MY1506, and MY1512 cells. Cells were grown in LB medium at 37°C, harvested at the early exponential phase by centrifugation at 4,000 × *g* for 10 min at 4°C, and washed with phosphate-buffered saline (PBS) three times. The SEM and TEM analyses were performed as previously described (69). For SEM and TEM analyses, images were collected using Hitachi SU8010 and HT7700 electron microscopes, respectively.

### Polysome analysis.

Polysome analysis was performed as described previously (70, 71), with minor modifications. Chloramphenicol was used to trap polysomes, and thus the *cat* gene was knocked out in suppressor cells. MG1655, MY1506Δ*cat*, and MY1512Δ*cat* strains were grown in LB medium at 37°C. Ribosomes were fractionated from cells by sucrose gradient centrifugation. When the OD_600_ of cells reached ∼0.3 to 0.4, polysomes were trapped by adding chloramphenicol to a final concentration of 0.1 mg ml^−1^. After incubation for 4 min, cells were rapidly cooled on ice and collected by centrifugation at 4,000 × *g* for 10 min at 4°C. Cell pellets were incubated in 1/50 volume of TKM buffer (20 mM Tris-HCl [pH 7.6], 60 mM KCl, 10 mM MgCl_2_, 20% [wt/vol] sucrose) with lysozyme (0.1 mg ml^−1^) for 20 min. The suspension was frozen in liquid nitrogen and then thawed slowly in a water bath at 30°C until melted. After three freeze-thaw cycles, a volume of TKM buffer containing 15 μl of deoxycholate (10%) and 10 μl of DNase I (1 mg ml^−1^) equal to 1/200 volume of the initial culture volume was added. The mixture was incubated on ice for ∼20 min (until the viscosity decreased). Lysates were then clarified by centrifugation at 18,000 × *g* for 10 min at 4°C. The extract concentration was estimated by measuring the *A*_260_. Between 25 and 100 *A*_260_ units of lysates was layered onto 10-to-40% (wt/wt) sucrose gradients and centrifuged at 21,000 × *g* for 3 h on a Beckman SW 41 Ti rotor at 4°C. Gradients were separated using the ÄKTA equipment, and their UV spectra were monitored.

### Sample preparation for proteomic analysis.

Whole-cell lysates from wild-type strain MG1655 and suppressor strains MY1506 and MY1512 were extracted as previously described (72), with modifications. Strains were grown in LB medium at 37°C and harvested at the mid-exponential growth phase by centrifugation at 6,000 × *g* for 10 min at 4°C. Cell pellets were then suspended in 100 mM Tris-HCl (pH 7.8) containing 2% (wt/vol) SDS and 100 mM dithiothreitol (DTT). Lysis was achieved by incubation at 99°C for 5 min. Cell debris and insoluble protein aggregates were removed by centrifugation at 16,000 × *g* for 60 min at 4°C, and the supernatants were used for analysis. The whole-cell lysates were processed according to the filter-aided sample preparation (FASP) protocol (73). The proteome of whole-cell lysates was analyzed through a data-dependent acquisition (DDA) mass spectrometry (MS) technique (74).

Inner membrane proteins of strains were isolated as described previously (35, 75). The cultures were harvested as described above. Cell pellets were resuspended in buffer A (50 mM Tris-HCl [pH 8.0], 20% [wt/vol] glycerol, 1 mM EDTA, 1 mM DTT, 50 μg ml^−1^ DNase I, 50 μg ml^−1^ RNase, 2.5 mM MgCl_2_, 1 mM phenylmethylsulfonyl fluoride [PMSF], 50 μg ml^−1^ lysozyme) at 4°C. The concentration of KCl was brought to 150 mM before cell lysis. Cells were lysed in a French press at 16,000 lb/in^2^ at 4°C. The sample solution was diluted with buffer A containing 150 mM KCl, and then sediment and unbroken cells were removed via centrifugation. The supernatant was centrifuged at 120,000 × *g* at 4°C for 60 min with a Beckman type 70 Ti rotor. Pellets were resuspended in buffer B (50 mM Tris-HCl [pH 8.0], 150 mM KCl, 1 mM EDTA, 1 mM DTT), loaded on a five-step sucrose gradient (1.9, 1.7, 1.5, 1.3, and 1.1 M sucrose in buffer B, pH 8.0), and centrifuged at 75,000 × *g* at 4°C for 14 h in a swing-out rotor (SW 32 Ti). The inner membrane fraction was collected using a syringe and diluted with 50 mM Tris-HCl (pH 8.0) to obtain a sucrose concentration less than 10%, and the membrane was reharvested via ultracentrifugation. Inner membrane pellets were then washed three times with buffer C (50 mM Tris-HCl [pH 8.0], 50 mM KCl, 5 mM MgCl_2_) at 120,000 × *g* at 4°C for 90 min. The inner membrane pellets were resuspended in an equal volume of ice-cold 100 mM Na_2_CO_3_ and agitated on ice for 1 h. The solution was ultracentrifuged and suspended in buffer C. Finally, the membrane solution was placed on top of a sucrose cushion solution (0.2 M sucrose, 50 mM Tris-HCl [pH 8.0], 50 mM KCl), and the membrane was pelleted via ultracentrifugation at 100,000 × *g* at 4°C for 30 min in a swing-out rotor (SW 41 Ti). The membrane pellets were resuspended in buffer B and stored at −80°C until further use.

Inner membrane proteins were digested with trypsin by surface proteolysis (75). Briefly, an aliquot of inner membrane protein was diluted in 50 mM Tris-HCl (pH 8.0), 50 mM KCl, and 5 mM MgCl_2_, and the total protein content of the membrane was estimated by a bicinchoninic acid (BCA) protein assay (Pierce, Thermo Scientific). The 400 μg amount of inner membrane protein was adjusted to 4 ml using a 50 mM ammonium bicarbonate solution (ABS). Proteins were reduced by 5 mM Tris (2-carboxyethyl) phosphine (TCEP) for 30 min and alkylated with 10 mM iodoacetamide (IAA) for 45 min in the dark at room temperature. Samples were digested overnight with trypsin gold (Promega, Madison, WI) according to the manufacturer’s instructions and then acidified with trifluoroacetic acid (TFA) until pH <2 was reached. After digestion, the protein solution was collected by ultracentrifugation at 200,000 × *g* at 4°C for 30 min with a SW 41 Ti rotor and the supernatant was collected. The supernatant was desalted using StageTips C_18_ (Thermo Scientific, USA) according to the manufacturer’s instructions. After desalting, the peptide mixture was dried under vacuum during centrifugation and reconstituted into loading solution (0.1% formic acid, 5% acetonitrile) for a data-independent acquisition (DIA) method, sequential window acquisition of all theoretical spectra (SWATH) analysis (76).

### Proteomic Analysis by LC-MS/MS.

The generated tryptic peptides were directly delivered to a NanoLC-2D ultra system (Eksigent, USA) equipped with a TripleTOF 5600 mass spectrometer (Sciex, USA). Each sample was injected and analyzed three times. Peptides were trapped on a NanoLC trap column (Chromxp C_18_CL, 3 μm, 120 Å, 350 μm by 0.5 mm; Eksigent) and then eluted onto an analytical column (Chromxp C_18_CL, 3 μm, 120 Å, 75 μm by 150 mm; Eksigent) and separated by a 120-min gradient as follows: buffer B from 5.0% to 60% (buffer A, 2.0% acetonitrile, 98% H_2_O, 0.1% formic acid; buffer B, 98% acetonitrile, 2.0% H_2_O, 0.1% formic acid) at a flow rate of 300 nl min^−1^. Key parameters for MS in DDA and DIA analysis were as described below. Full-scan MS was performed in the positive ion mode with a nano-ion spray voltage of 2.5 kV from 350 to 1,500 (*m/z*). For DDA analysis, survey scans were acquired in 250 ms, and as many as 30 product ion scans (*m/z* 100 to 1,500) were collected if a threshold of 125 cps was exceeded and with a +2 to +5 charge state. For DIA quantification analysis, MS parameters were set to acquire an MS scan in the range of 400 to 1,250 Da, followed by 60 variable SWATH windows.

The raw data were processed and searched against E. coli MG1655 proteins registered in the UniProt database (Proteome ID UP000000625, 4,391 protein entries, version August 2019) (77) using ProteinPilot 4.5 software (Sciex, USA). Some important parameters in the paragon search algorithm in ProteinPilot were configured as follows: sample type, identification; Cys alkylation, MMTS; digestion, trypsin; instrument, TripleTOF 5600; search effort, thorough ID. Protein and peptide false discovery rate (FDR) analysis was performed in ProteinPilot, and the FDR was set to 1% for identification. The result of protein identification was also used as a library for SWATH quantification analysis. PeakView software 2.0 with SWATH was used to assign the correct peaks to correct peptides in the library. One to 10 peptides per protein were selected to be used in SWATH quantification (76). Peptide data were then normalized by the median scale normalization (MedScale) method (78).

### Measurement of translation elongation rate.

The translational elongation rate was measured as previously described (33, 79), with modifications. As the elongation rate assay was based on the LacZ induction assay, the *lacZ* genes in strains MG1655, MY1506, and MY1512 were all knocked out. HDB51 is a LacZ-deficient E. coli strain (65). Chloramphenicol was used to inhibit elongation in this assay, so the *cat* gene in suppressor cells was also deleted. The seed culture and preculture were prepared as described by Zhu and coauthors (79), except for the HDB51 strain. For seed culture, the HDB51 strain was grown in LB containing 0.2% arabinose at 37°C for several hours. To generate SRP-positive (SRP^+^) or SRP-negative (SRP^−^) cells, the culture was collected and washed with fresh MOPS minimal medium (80) and then cultivated in MOPS medium with 0.2% arabinose or 0.2% glucose overnight at 37°C or 25°C as a preculture. For MG1655ΔlacZ and SRP^+^ strains, the experimental culture was performed with an initial OD_600_ of 0.01 to 0.02. For the MY1506Δ*lacZ*Δ*cat*, MY1512Δ*lacZ*Δ*cat*, and SRP^−^ strains, experimental cultures were performed with an initial OD_600_ of 0.04 to 0.05. All strains were grown to an OD_600_ of 0.4 to 0.5 in the identified MOPS medium. A final concentration of 5 mM IPTG was added to the cultures. Every 10 or 20 s, 1 ml of culture was collected and pipetted into precooled Eppendorf tubes containing 10 μl chloramphenicol (34 mg ml^−1^), snap frozen in liquid nitrogen, and stored at −80°C before the subsequent LacZ assay. The procedure of the LacZ assay was the same as described by Zhu et al. (79).

LacZα induction kinetics were measured as previously described (33, 79). To estimate the translational elongation rate, the time cost of initiation steps (*T*_init_) and the synthesis times of LacZα (*T*_α_) and LacZα-fused protein (*T*_total_) were calculated by the LacZ induction assay. *T*_α_ was estimated by using a flat-line fit of the LacZα induction curve. The *x* coordinate of the intersection point is *T*_α_. *T*_total_ was estimated by plotting the square root of a new synthesized protein with the induction time. The *x* intercept of the obtained linear line is the *T*_total_. The initiation time was calculated as *T*_init_ = *T*_α_ – {90/[*L*/(*T*_total_ – *T*_α_)]}, where 90 is the 90-residue-long LacZα fragment and *L* is the length of the target protein (containing 10 aa linker). The time delay (*T*_delay_) in initiation of suppressor cells was calculated as *T*_delay_ = *T*_init_ (suppressor cells) – *T*_init_ (wild-type cells), when cells were grown at the same growth rate. Then the elongation time of the LacZα-fused protein was corrected by subtracting the initiation time (*T*_init_) from the synthesis time of the LacZα-fused protein (*T*_total_). Then, the elongation rate (ER) was calculated as (*L* + 90)/(*T*_total_ – *T*_init_).

### Estimation of translational initiation rate.

The initiation rate has not yet been effectively estimated by experimental approaches. Computational models of translation were used to predict the initiation rate (81–83). The ribosome flow model (RFM) is a computational model for translation elongation (84). Under the assumption that translational elongation rates are nearly constant in some cases, the RFM becomes the homogeneous ribosome flow model (HRFM) (34). This model includes two parameters: the initiation rate λ and the constant elongation rate λ*_c_* (81, 83). The translation rate *R* equals *R*(λ, λ*_c_*). The initiation rate λ was estimated by the formulas below:

$$\text{λ}=b(\text{1}-\sqrt{\omega_{\text{1}}{\text{/(ω}}_{\text{1}}{+\omega }_{\text{2}}\text{)}}{\text{)/ω}}_{\text{2}}$$

$${\text{λ}}_{c}=b/\sqrt{\omega_{\text{1}}{\text{(ω}}_{\text{1}}{+\omega }_{\text{2}}\text{)}}$$

$$R=b(\text{2}\omega_{\text{1}}+\omega_{\text{2}}-2\sqrt{\omega_{\text{1}}\left(\omega_{\text{1}}{+\omega }_{\text{2}}\right)}{\text{)/ω}}_{\text{2}}^{\text{2}}$$

given the parameter $\omega_{\text{1}},\omega_{\text{2}},b>0$ and parameters λ and λ*_c_* are subjected to the constraints:

$$\omega_{\text{1}}{\text{λ}}_{c}+\omega_{\text{2}}\text{λ}\leq b$$

$${\text{λ}}_{c},\lambda \geq 0$$

In the HRFM model, the elongation rate λ*_c_* equals $\bar{\lambda_{c}}$ (aa s^−1^)/11 (sites s^−1^) (83), since each ribosome occupies about 11 codons in E. coli (85). The translation rate *R* in E. coli is calculated by *R* equals $\bar{R}$(aa s^−1^)/336 (proteins mRNA^−1^ s^−1^) (83), since the average length of proteins in E. coli is 336 amino acids (86). And $\bar{R}$ (aa s^−1^) equals (*L* + 90)/(*T*_total_ – 9), which parallels the calculation of the elongation rate. *L* represents the length of a target protein (containing 10 aa linker). We assumed that the average lapse of time before the initiation of translation is 9 s (87).

### SDS-PAGE and immunoblotting.

Targeting of membrane proteins was monitored by whole-cell immunoblotting as described previously (42, 88), with minor modifications. E. coli MG1655Δ*lacZ*, HDB51 (SRP^+^ and SRP^−^), MY1506Δ*lacZ*Δ*cat*, and MY1512Δ*lacZ*Δ*cat* strains, which containing derivatives of pJH29, were incubated at 37°C in LB medium supplemented with 20 μg ml^−1^ chloramphenicol. Protein induction was carried out when MG1655Δ*lacZ* was grown to an OD_600_ of 0.8 to 1.0, with 0.02 mM IPTG. Cultures were harvested when the OD_600_ reached ∼2.0. For the HDB51 strain, its overnight culture was washed with the fresh LB medium twice and diluted into the fresh LB with or without 0.2% arabinose to generate SRP^+^ and SRP^−^cells, respectively. For the SRP^+^ strain, protein expression was inducted as described for MG1655Δ*lacZ*. As demonstrated in previous works, the protein expression level of Ffh was completely abolished when cells were cultivated for 2 h (23). SRP^−^ cells were cultivated for 2.5 to 3 h until the OD_600_ reached 0.6 to 0.7, and then protein expression was induced with 0.02 mM IPTG. For Ffh inhibition, 0.2% glucose was added 2 h before harvesting cells. The culture was harvested when the OD_600_ reached ∼1.5. MY1506Δ*lacZ*Δ*cat* and MY1512Δ*lacZ*Δ*cat* cells were grown to an OD_600_ of 0.55 to 0.65, induced with 0.02 mM IPTG, and then harvested when the OD_600_ reached ∼1.5. For the SRP^−^ strain grown at a low temperature, 25°C, the protein expression was induced with 0.02 mM IPTG when the OD_600_ reached 0.45 to 0.55 and then cells were harvested when the OD_600_ reached ∼1.0. When the SRP^−^ strain was cultivated in LB medium containing 50 μg ml^−1^ kasugamycin (Ksg), protein expression was inducted as described for the SRP^−^ strain at 37°C, but the Ksg was added 20 min earlier, before the induction. The culture was harvested when the OD_600_ reached ∼1.2. Whole-cell samples were resuspended in ∼150 μl of ice-cold solution (50 mM KP_i_ [pH 7.2], 1 mM MgSO_4_, 10% [wt/vol] glycerol, 1 mM PMSF, trace amounts of DNase I). Cells were disrupted by glass beads with vigorous agitation (42). Fifty microliters of solubilization buffer was then added to 50 μl of whole-cell lysates. Samples were incubated at 37°C for 5 min (88). Whole-cell lysates were analyzed by SDS-PAGE using 12% or 15% gels, followed by immunoblotting. Proteins were transferred to PVDF membranes, probed with an anti-His tag antibody, and detected by horseradish peroxidase (HRP)-conjugated second antibody. Detection was performed using a DAB substrate kit (Thermo Scientific, USA). Blots were quantified using Image Lab 5.0 software (Bio-Rad).
