# Supplementary material for: Signal Recognition Particle Suppressor Screening Reveals the Regulation of Membrane Protein Targeting by the Translation Rate
Source: mBio. 2021 Jan 12;12(1):e02373-20. doi: 10.1128/mBio.02373-20 (PMC7844537; doi:10.1128/mBio.02373-20)
Supplement: FIG S1 [file mBio.02373-20-sf001.pdf]

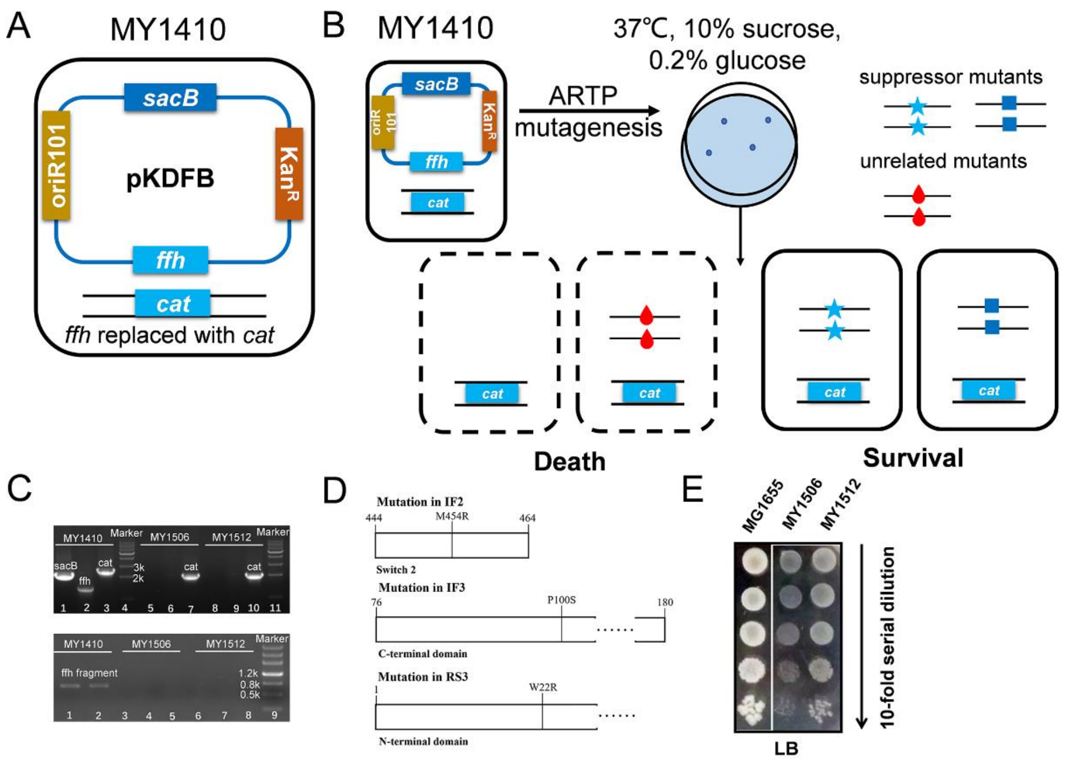

FIG S1 Isolation of SRP suppressors. (A) Construction of the MY1410 strain for screening suppressors. A temperature-sensitive plasmid pKDFB containing the *ffh* gene and a counterselection marker gene *sacB* was used to rescue Ffh-deletion cells. (B) Suppressor screening. The cells without suppressors were killed by any of the following conditions: 37°C, 10% (w/v) sucrose and 0.2% (w/v) glucose. (C) Agarose gel electrophoresis of colony PCR. Samples from strain MY1410 were used as controls. (D) Localization of suppressors in protein sequences. (E) Plating assay of wild-type MG1655 and candidate suppressor MY1506 and MY1512 strains.
