# Supplementary material for: Signal Recognition Particle Suppressor Screening Reveals the Regulation of Membrane Protein Targeting by the Translation Rate
Source: mBio. 2021 Jan 12;12(1):e02373-20. doi: 10.1128/mBio.02373-20 (PMC7844537; doi:10.1128/mBio.02373-20)
Supplement: FIG S2 [file mBio.02373-20-sf002.pdf]

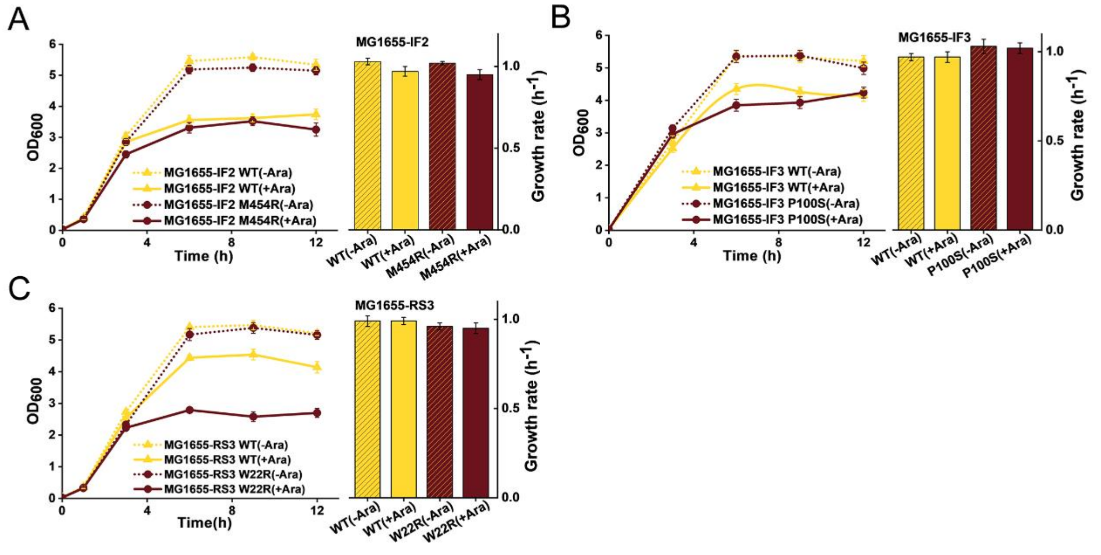

FIG S2 Growth assays of MG1655 strain expression of wild-type (WT) and suppressor proteins. Protein expression was induced by the addition of arabinose (+Ara). Non-induced cells were cultured without arabinose (−Ara). (A) Growth curves and growth rates of the MG1655 strain overproducing IF2 WT and suppressor IF2 M454R. (B) Growth curves and growth rates of the MG1655 strain overproducing IF3 WT and suppressor IF3 P100S. (C) Growth curves and growth rates of the MG1655 strain overproducing RS3 WT and suppressor RS3 W22R. The strains had approximately the same growth rate of 1.0 h^{-1}, but the OD_{600} of the induced cells decreased during entry into the stationary phase compared with that of the non-induced cells. Solid or short dotted curves are the mean of three independent samples and error bars represent the standard error of the mean (SEM) of the samples. Growth rates were calculated from the exponential growth phase. All growth rates shown represent the mean growth rates from three biological replicates, and the error bars represent the SEM value.
