# Supplementary material for: Signal Recognition Particle Suppressor Screening Reveals the Regulation of Membrane Protein Targeting by the Translation Rate
Source: mBio. 2021 Jan 12;12(1):e02373-20. doi: 10.1128/mBio.02373-20 (PMC7844537; doi:10.1128/mBio.02373-20)
Supplement: FIG S3 [file mBio.02373-20-sf003.pdf]

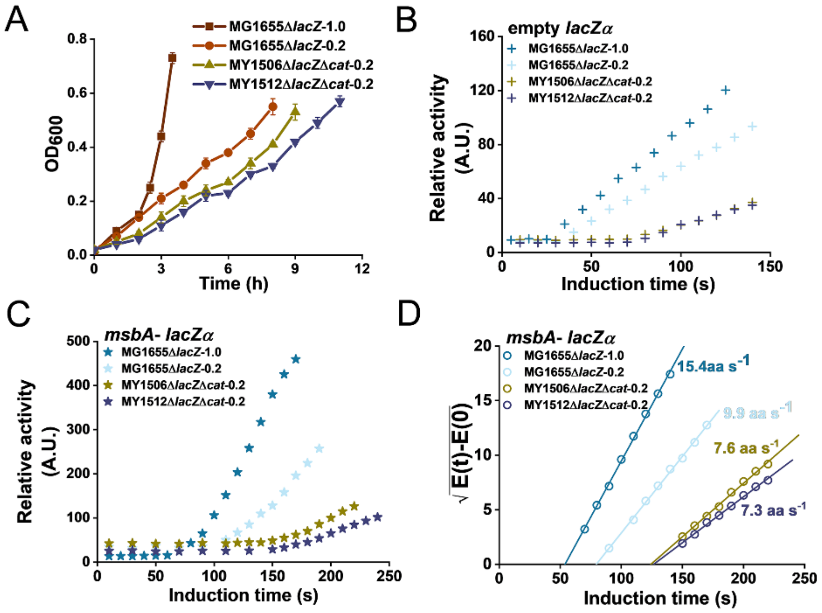

FIG S3 Measurement of the translation elongation rates of wild-type and suppressor cells. (A) Growth curves of strains in different MOPS media. The growth rates of MG1655Δ*lacZ*, MY1506Δ*lacZ*Δ*cat* and MY1512Δ*lacZ*Δ*cat* grown in Glucose + cAA medium were approximately 1.0 h^{-1}, 0.2 h^{-1} and 0.2 h^{-1}, respectively. The growth rate of MG1655Δ*lacZ* grown in Sorbitol + NH_4Cl medium was approximately 0.2 h^{-1}(Table S1A). Solid curves are the mean of three independent biological replicates, and the error bars represent the SEM values. (B) Calibration of the time cost of initiation steps (T_{init}) by measuring the induction kinetics of the empty LacZα fragment. (C) The induction curves of the LacZα-fused protein MsbA-LacZα. (D) Schleif plots of the MsbA-LacZα protein plotted against the induction time. Schleif plots were repeated for three times, and one typical result shown here.
