# Supplementary material for: Signal Recognition Particle Suppressor Screening Reveals the Regulation of Membrane Protein Targeting by the Translation Rate
Source: mBio. 2021 Jan 12;12(1):e02373-20. doi: 10.1128/mBio.02373-20 (PMC7844537; doi:10.1128/mBio.02373-20)
Supplement: TABLE S1 [file mBio.02373-20-st001.pdf]

TABLE S1 File containing the cell growth in MOPS media and properties of first transmembrane domains. (A) Growth rates of cells under different nutrient conditions. Growth rates were calculated from the corresponding growth curves that are shown in Fig. S3 and Fig. S4. All values are expressed as the mean ± SEM. Data shown are related to Fig. 2E and F and Fig. 4A to D. (B) First transmembrane domain (TMD1) properties analysis. Data shown are related to Fig. 5E.

TABLE S1A

**Strain**	**Nutrient condition**	**Growth rate (h^{-1})**	**T_{int}**	**T_{delay}**
MG1655Δ*lacZ*	Glucose + cAA<br>0.2% glucose + 0.2% casamino acids	1.10 ± 0.04	9.7±0.2	–
MG1655Δ*lacZ*	Sorbitol + NH_4Cl<br>0.1% sorbitol and 10 mM NH_4Cl	0.19 ± 0.03	10.9±0.3	–
MY1506Δ*lacZ*Δ*cat*	Glucose + cAA<br>0.2% glucose + 0.2% casamino acids	0.20 ± 0.02	34.2±0.4	23.3±0.4[a]
MY1512Δ*lacZ*Δ*cat*	Glucose + cAA<br>0.2% glucose + 0.2% casamino acids	0.19 ± 0.02	39.8±0.4	28.9±0.4[a]
SRP^+	Arabinose + fructose + cAA<br>0.2% arabinose + 0.2% fructose + 0.4% casamino acids	1.08 ± 0.11	9.9±0.1	–
SRP^-	Fructose + cAA<br>0.2% fructose + 0.15% casamino acids	0.22± 0.02	11.1±0.4	–
SRP^-(25°C)	Fructose + cAA<br>0.2% fructose + 0.15% casamino acids	0.18±0.01	12.1±0.1	1.0±0.4[b]
SRP^-(+Ksg)	Fructose + cAA<br>0.2% fructose + 0.15% casamino acids	0.12±0.01	17.4±0.3	6.3±0.4[b]

[a]The time delay in initiation relative to MG1655Δ*lacZ* cell grown at ~0.2 h^{-1}.

[b]The time delay in initiation relative to SRP^- cell grown at 37°C.

TABLE S1B

Category	Presence of category in TMD1 sequence in %	
	Targeted	Untargeted
average Gly content	6	7
average Ser content	4	3
average ALA content	12	10
average Thr content	6	5
average Val content	11	11
average Asp content	0	0
average Asn content	0	1
average Leu content	23	21
average Ile content	11	12
average Glu content	0	1
average Gln content	1	1
average Tyr content	3	2
average Phe content	7	10
average His content	1	1
average Pro content	2	2
average Met content	3	4
average Trp content	4	2
average Lys content	1	2
average Cys content	1	1
average Arg content	3	2
average polar	15	14
average charged	6	6
average Aramatics	14	15
average Aliphatics	49	48
average Hydrophobics	80	80
average Leu:Ala ratio	2	2
