# Supplementary material for: Signal Recognition Particle Suppressor Screening Reveals the Regulation of Membrane Protein Targeting by the Translation Rate
Source: mBio. 2021 Jan 12;12(1):e02373-20. doi: 10.1128/mBio.02373-20 (PMC7844537; doi:10.1128/mBio.02373-20)
Supplement: FIG S4 [file mBio.02373-20-sf004.pdf]

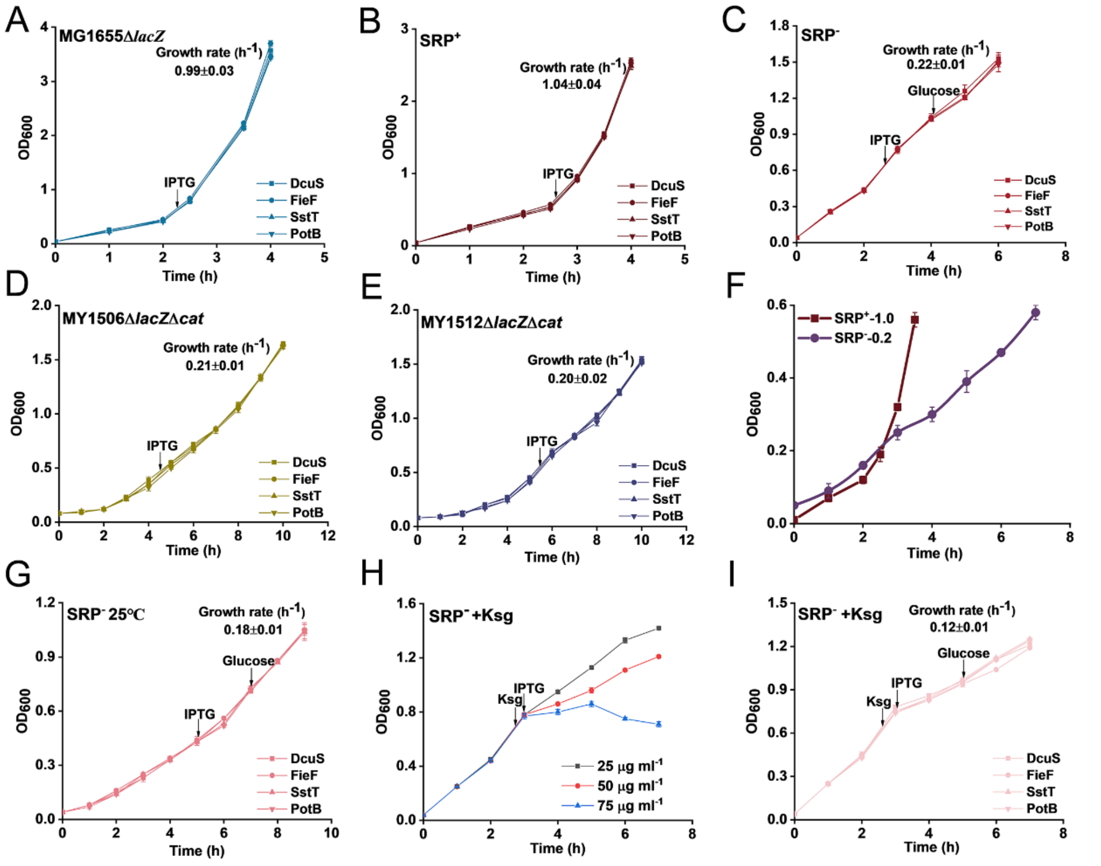

FIG S4 Growth curves of cells for protein targeting and translation elongation rate assays. (A-E) Growth curves for the expression of GFP-His_8 fused proteins in MG1655Δ*lacZ* (A), SRP^+(B), SRP^-(C), MY1506Δ*lacZ*Δ*cat* (D) and MY1512Δ*lacZ*Δ*cat* (E). SRP^+, Ffh expression in the HDB51 strain grown in addition of arabinose; SRP^-, Ffh depletion in the HDB51 strain grown in addition of glucose. (F) Growth curves of HDB51 cells in different MOPS media. The growth rate of SRP^+ cells grown in Arabinose + fructose + cAA was approximately 1.0 h^{-1}. The growth rates of SRP^- cells grown in Fructose + cAA medium was approximately 0.2 h^{-1} (Table S1A). (G) Growth curves for expression of GFP-His_8 fused proteins in SRP^- cells at 25°C. (H) Growth curves for the expression of DcuS-GFP-His_8 in SRP^- cells that grown in LB medium supplemented with 25, 50 and 75 μg ml^{-1} kasugamycin (Ksg). A high

concentration of Ksg (75 μg ml^{-1}) caused cell death. (I) Growth curves for the expression of GFP-His_8 fused proteins in SRP^- cells grown in LB medium supplemented with 50 μg ml^{-1} Ksg. The solid curves are the mean of three independent biological replicates, and the error bars represent the SEM values. Growth rates were calculated from the exponential growth phase. All values are expressed as the mean ± SEM.
