# Supplementary material for: Signal Recognition Particle Suppressor Screening Reveals the Regulation of Membrane Protein Targeting by the Translation Rate
Source: mBio. 2021 Jan 12;12(1):e02373-20. doi: 10.1128/mBio.02373-20 (PMC7844537; doi:10.1128/mBio.02373-20)
Supplement: FIG S5 [file mBio.02373-20-sf005.pdf]

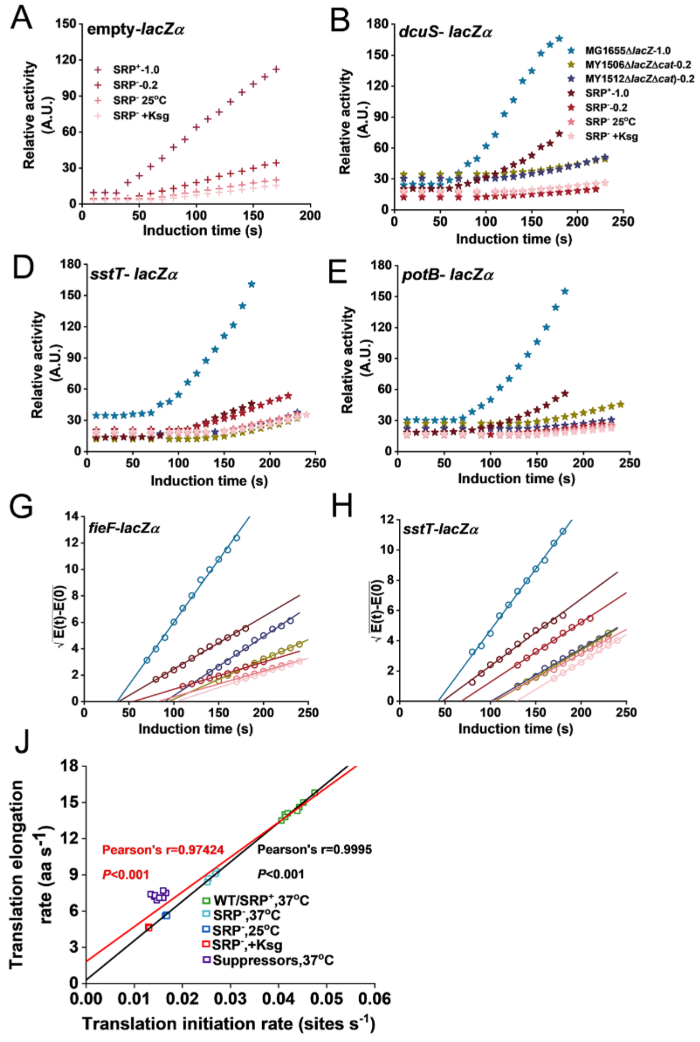

FIG S5 Measurement of translational elongation rates and the correlations between initiation and elongation in different cells. (A) Calibration of the time cost of initiation steps (T_{init}) of different cells by measuring the induction kinetics of the empty LacZα fragment. (B-E) Induction kinetics of the DcuS-LacZα protein (B), FieF-LacZα protein (C), SstT-LacZα protein (D) and PotB-LacZα protein (E). The MG1655Δ*lacZ*, MY1506Δ*lacZ*Δ*cat*, MY1512Δ*lacZ*Δ*cat* and SRP^+ strains were grown at 37°C. The SRP^- strain was grown at 37°C or 25°C and treated with kasugamycin (Ksg). (F-I) Schleif plot of the DcuS-LacZα protein (F), FieF-LacZα protein (G), SstT-LacZα protein (H) and PotB-LacZα protein (I). The Schleif plots from panels F to I were

repeated three times, and one typical result is shown here. (J) Significant correlation existed between translation initiation and elongation in different cells. After the removal of suppressor cells, this correlation was stronger.
