# Supplementary material for: Signal Recognition Particle Suppressor Screening Reveals the Regulation of Membrane Protein Targeting by the Translation Rate
Source: mBio. 2021 Jan 12;12(1):e02373-20. doi: 10.1128/mBio.02373-20 (PMC7844537; doi:10.1128/mBio.02373-20)
Supplement: FIG S6 [file mBio.02373-20-sf006.pdf]

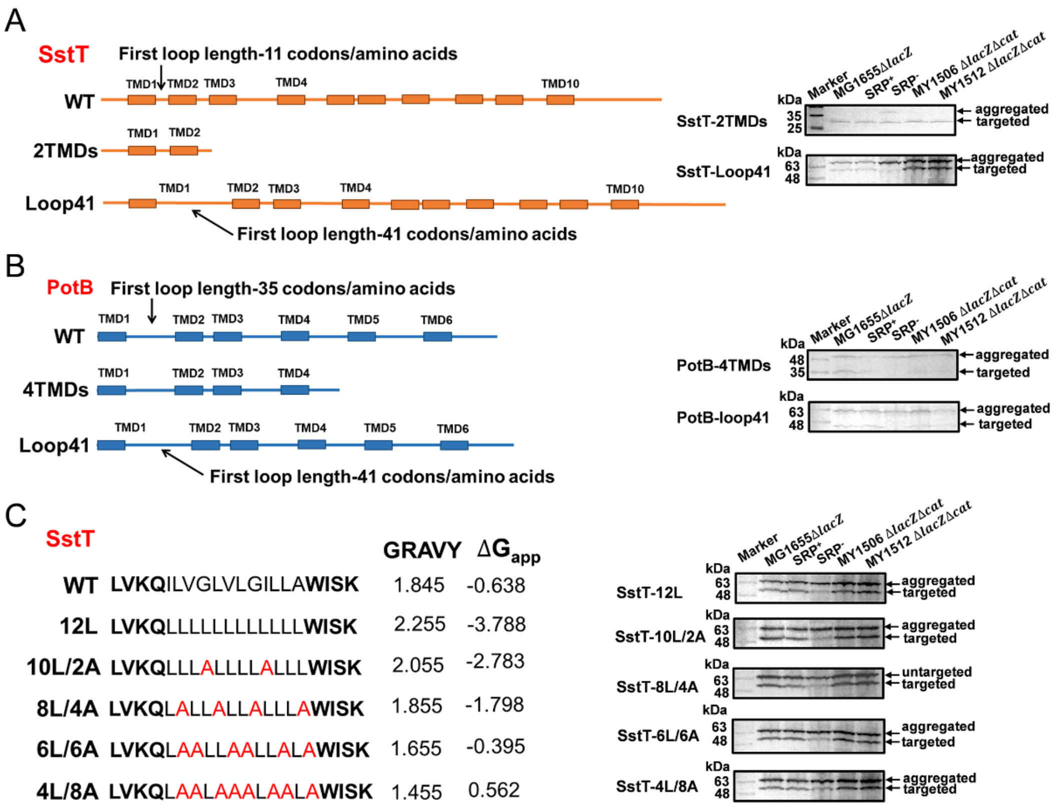

FIG S6 Immunoblotting analysis of several membrane protein variants. (A-B) Detection of targeted and aggregated GFP-fused SstT (A) and PotB (B) variants. Left panel: Secondary structures of wild-type and mutant proteins. Right panel: immunoblotting. (C) Detection of targeted and aggregated GFP-fused SstT variants in which TMD1 had different hydrophobicity or ΔG_{app} values. Left panel: Secondary structures of wild-type and mutant proteins. The grand averages of hydropathy (GRAVY) and ΔG_{app} values are shown. Right panel: immunoblotting.
