# Supplementary material for: Signal Recognition Particle Suppressor Screening Reveals the Regulation of Membrane Protein Targeting by the Translation Rate
Source: mBio. 2021 Jan 12;12(1):e02373-20. doi: 10.1128/mBio.02373-20 (PMC7844537; doi:10.1128/mBio.02373-20)
Supplement: FIG S7 [file mBio.02373-20-sf007.pdf]

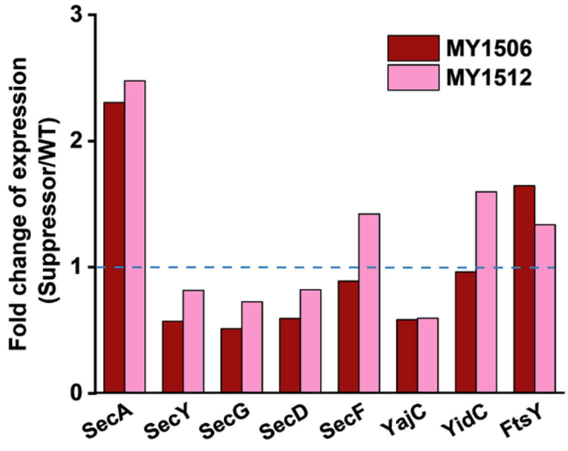

FIG S7 The fold-change in the expression of transporter genes in suppressor cells relative to those in wild-type cells. The dashed line indicates the expression level of proteins in wild-type cells.
